# Brief Sensory Deprivation Triggers Cell Type-Specific Structural and Functional Plasticity in Olfactory Bulb Neurons

**DOI:** 10.1523/JNEUROSCI.1606-20.2020

**Published:** 2021-03-10

**Authors:** Elisa Galliano, Christiane Hahn, Lorcan P. Browne, Paula R. Villamayor, Candida Tufo, Andres Crespo, Matthew S. Grubb

**Affiliations:** ^1^Centre for Developmental Neurobiology, Institute of Psychiatry, Psychology & Neuroscience, King's College London, London, SE1 1UL, United Kingdom; ^2^Department of Physiology, Development and Neuroscience, University of Cambridge, Cambridge, CB2 3DY, United Kingdom

**Keywords:** axon initial segment, dopamine, olfaction, plasticity, sensory deprivation

## Abstract

Can alterations in experience trigger different plastic modifications in neuronal structure and function, and if so, how do they integrate at the cellular level? To address this question, we interrogated circuitry in the mouse olfactory bulb responsible for the earliest steps in odor processing. We induced experience-dependent plasticity in mice of either sex by blocking one nostril for one day, a minimally invasive manipulation that leaves the sensory organ undamaged and is akin to the natural transient blockage suffered during common mild rhinal infections. We found that such brief sensory deprivation produced structural and functional plasticity in one highly specialized bulbar cell type: axon-bearing dopaminergic neurons in the glomerular layer. After 24 h naris occlusion, the axon initial segment (AIS) in bulbar dopaminergic neurons became significantly shorter, a structural modification that was also associated with a decrease in intrinsic excitability. These effects were specific to the AIS-positive dopaminergic subpopulation because no experience-dependent alterations in intrinsic excitability were observed in AIS-negative dopaminergic cells. Moreover, 24 h naris occlusion produced no structural changes at the AIS of bulbar excitatory neurons, mitral/tufted and external tufted cells, nor did it alter their intrinsic excitability. By targeting excitability in one specialized dopaminergic subpopulation, experience-dependent plasticity in early olfactory networks might act to fine-tune sensory processing in the face of continually fluctuating inputs.

**SIGNIFICANCE STATEMENT** Sensory networks need to be plastic so they can adapt to changes in incoming stimuli. To see how cells in mouse olfactory circuits can change in response to sensory challenges, we blocked a nostril for just one day, a naturally relevant manipulation akin to the deprivation that occurs with a mild cold. We found that this brief deprivation induces forms of axonal and intrinsic functional plasticity in one specific olfactory bulb cell subtype: axon-bearing dopaminergic interneurons. In contrast, intrinsic properties of axon-lacking bulbar dopaminergic neurons and neighboring excitatory neurons remained unchanged. Within the same sensory circuits, specific cell types can therefore make distinct plastic changes in response to an ever-changing external landscape.

## Introduction

One way that animals can ensure appropriate behavioral choices when faced with an ever-changing environment is to alter the way they process sensory inputs. To implement such adaptive control at the level of neuronal networks, there exists a huge range of cellular mechanisms of neuronal plasticity. These include structural changes in neuronal morphology, functional changes of synaptic strength, and/or modulation of intrinsic excitability (Citri and Malenka, 2008; Kullmann et al., 2012; Wefelmeyer et al., 2016; Brzosko et al., 2019; Debanne et al., 2019; Roy et al., 2020). This extensive repertoire also includes a form of structural plasticity tightly linked with changes in neuronal excitability: plasticity of the axon initial segment (AIS).

Structurally, the AIS is a subcellular zone located in the proximal portion of the axon, where an intricate arrangement of cytoskeletal and scaffolding proteins anchors a membrane-bound collection of signaling molecules, receptors, and ion channels (Leterrier, 2018; Vassilopoulos et al., 2019). Functionally, the AIS serves two key roles: maintenance of dendritic/axonal polarity (Hedstrom et al., 2008; Hamdan et al., 2020) and initiation of action potentials (APs) (Bean, 2007; Kole et al., 2007). Plastically, the AIS has been proven capable of changing its structure in terms of length, distance from the soma, and/or molecular content (Grubb and Burrone, 2010; Kuba et al., 2010, 2015; Lezmy et al., 2017; Ding et al., 2018).

How is AIS plasticity driven by changes in neuronal activity? *In vitro*, elevated activity can cause the AIS of excitatory neurons to relocate distally or to decrease in length, structural changes that are usually associated with decreased functional excitability (Grubb and Burrone, 2010; Evans et al., 2013, 2015; Muir and Kittler, 2014; Chand et al., 2015; Horschitz et al., 2015; Wefelmeyer et al., 2015; Lezmy et al., 2017; Sohn et al., 2019). *In vivo*, activity-dependent structural AIS plasticity has been observed in excitatory neurons, usually induced by manipulations that are long in duration and/or involve damage to peripheral sensory organs (Kuba et al., 2010; Gutzmann et al., 2014; Akter et al., 2020; Pan-Vazquez et al., 2020; but see Jamann et al., 2021). But is AIS plasticity a prerogative of excitatory neurons, or is it also included in the plasticity toolkit of inhibitory cells? We previously found that, *in vitro*, inhibitory dopaminergic (DA) interneurons in the olfactory bulb (OB) are capable of bidirectional AIS plasticity, inverted in sign with respect to their excitatory counterparts: their AIS increases in length and relocates proximally in response to chronic depolarization, and shortens when spontaneous activity is silenced (Chand et al., 2015). Together, these studies begin to paint a picture of how different cell types respond to changes in incoming activity levels by initiating distinct plastic structural changes at their AIS. However, many key questions remain unanswered. Are more physiological, minimally invasive sensory manipulations sufficient to induce AIS plasticity *in vivo*? In the intact animal, can AIS plasticity occur over more rapid timescales? And do excitatory and inhibitory neurons in sensory circuits respond to such brief and naturally relevant sensory manipulation with similar levels of AIS plasticity?

To address these questions, we interrogated circuitry in the mouse OB responsible for the earliest steps in odor processing (Shepherd, 2005). At just one synapse away from the sensory periphery, activity in the OB can be readily and reliably altered by physiologically relevant alterations in sensory experience (Coppola, 2012). In our case, this was achieved by unilaterally plugging a nostril for just one day, a minimally invasive manipulation that effectively mimics the sensory disturbance associated with common respiratory infections, without damaging the olfactory sensory epithelium (Fokkens et al., 2012). We found that such brief sensory deprivation produced structural and functional intrinsic plasticity in axon-bearing DA neurons in the bulb's glomerular layer (Chand et al., 2015; Galliano et al., 2018). By targeting excitability in one specialized DA subpopulation, experience-dependent plasticity in early olfactory networks might act to fine-tune sensory processing in the face of continually fluctuating inputs.

## Materials and Methods

### 

#### 

##### Animals

We used mice of either sex and housed them under a 12 h light-dark cycle in an environmentally controlled room with free access to water and food. WT C57BL/6 mice (Charles River) were used either as experimental animals, or to backcross each generation of transgenic animals. The founders of our transgenic mouse lines, DAT^IRES^*^cre^* (B6.SJL-*Slc6a3^tm1.1(cre)Bkmn^*/J, Jax stock #006660) and Ai9 (B6.Cg–*Gt(ROSA)26Sor^tm9(CAG-tdTomato)Hze^*/J; Jax stock #007909), were purchased from the Jackson Laboratory. All experiments were performed between postnatal days (P) 21 and 35. All experiments were performed at King's College London under the auspices of United Kingdom Home Office personal and project licences held by the authors.

##### Sensory manipulation

To perform unilateral naris occlusion, mice were briefly anesthetized (<5 min) with isoflurane. In the occluded (Occl) group, a custom-made ∼5 mm Vaseline-lubricated plug, constructed by knotting suture (Ethilon polymide size 6, nonabsorbable suture, Ethicon) around a piece of unscented dental floss and pulled through the lumen of PTFE tubing with an outer diameter of 0.6 mm and inner diameter of 0.3 mm (VWR, catalog #S1810-04) (see Cummings et al., 2014), was inserted into the right nostril where it remained for 24 h. Only the right OB was then used for experiments. At the termination of each experiment, *post hoc* visual observation of the nasal cavity was always performed to ensure that the plug had remained in place. The few mice where the plug could not be found were not used for experiments. All control (Ctrl) animals were gender- and age-matched mice left unperturbed in their home cage. For both Ctrl and Occl groups, only right bulbs were analyzed.

##### Immunohistochemistry

Mice were anesthetized with an overdose of pentobarbital and then perfused with 20 ml PBS with heparin (20 units/ml), followed by 20 ml of 1% PFA (TAAB Laboratories; in 3% sucrose, 60 mm PIPES, 25 mm HEPES, 5 mm EGTA, and 1 mm MgCl_2_; this relatively weak fixative solution facilitates staining for AIS-localized proteins, especially ankyrin-G [AnkG]).

To expose the olfactory epithelia, the rostral half of the calvaria (anterior to the bregma) and the nasal bone were removed, and the samples were first postfixed overnight (4°C) and then placed in 0.25 m EDTA (Invitrogen AM9261) in PBS at 4°C for 3 d for decalcification. After overnight cryoprotective treatment with 30% sucrose (Sigma Millipore, S9378), they were then embedded in OCT (VWR Chemicals, 00411243), frozen in liquid nitrogen, and sliced on a cryostat (Leica Microsystems, CM 1950) into 20 µm slices.

The OBs were dissected and postfixed in 1% PFA for 2-7 d, then embedded in 5% agarose, and sliced at 50 µm using a vibratome (VT1000S, Leica Microsystems). For experiments that aimed at comparing intensity of staining across mice, we co-embedded the bulbs of 1 Ctrl and 1 Occl mouse in a large agarose block (“set”); and from then forward, we processed them as a unit (Vlug et al., 2005). To assess the suitability of the co-embedding strategy and the variability of staining intensity between unperturbed animals, a subset of OBs from Ctrl mice were processed together: in the same agarose block, the right and left OB from one Ctrl mouse (Mouse 1) were co-embedded with the right OB from a second Ctrl mouse (Mouse 2).

Free-floating slices or sets were washed with PBS and incubated in 5% normal goat serum in PBS/Triton/azide (0.25% Triton, 0.02% azide) for 2 h at room temperature. They were then incubated in primary antibody solution (in PBS/Triton/azide; Table 1) for 2 d at 4°C.

**Table 1.** Primary antibodies used

Target	Host	Supplier	Dilution
TH	Rabbit	Millipore	1:500
TH	Mouse	Millipore	1:500
TH	Chicken	Abcam	1:250
AnkG	Mouse 2a	NeuroMab	1:500
AnkG	Mouse 2b	NeuroMab	1:500
AnkG	Mouse 1	NeuroMab	1:500
CCK	Rabbit	Immunostar	1:200
Neurofilament H nonphosphorylated (SMI-32)	Mouse	Biolegend	1:1000
cFos	Mouse	Santa Cruz Biotechnology	1:500
pS6	Rabbit	Cell Signaling	1:400
OMP	Goat	Wako	1:1000
Cleaved caspase-3	Rabbit	Cell Signaling Technology	1:1000

Slices were then washed 3 times for 5 min with PBS, before being incubated in secondary antibody solution (species-appropriate, Invitrogen AlexaFluor; 1:1000 in PBS/Triton/azide) for 3 h at room temperature. After washing in PBS, slices were either directly mounted on glass slides, Menzel-Gläser) with MOWIOL-488 (Calbiochem), or first underwent additional counterstaining steps with NucRed Live 647 (Invitrogen, R37106) at room temperature for 25 min to visualize cell nuclei, or with 0.2% Sudan black in 70% ethanol at room temperature for 3 min to minimize autofluorescence. Unless stated otherwise, all reagents were purchased from Sigma Millipore.

##### Fixed-tissue imaging and analysis

All images were acquired with a laser scanning confocal microscope (Carl Zeiss, LSM 710) using appropriate excitation and emission filters, a pinhole of 1 AU and a 40× oil immersion objective. Laser power and gain were set to either prevent signal saturation in channels imaged for localization analyses, or to permit clear delineation of neuronal processes in channels imaged for neurite identification (*e.g*., TH, SMI-32, cholecystokinin [CCK]). All quantitative analysis was performed with Fiji (ImageJ) by experimenters blind to group identity.

For olfactory epithelium (OE) analysis, four images were acquired from consistently positioned septal and dorsomedial regions of interest (ROIs) within each section, with a 1× zoom (0.415 µm/pixel), 512 × 512 pixels, and in *z* stacks with 1 µm steps. OE thickness was measured on single plane images by drawing a straight line, parallel to olfactory sensory neuron (OSN) dendrites, from the lamina propria to the tips of the OSN dendrites (visualized with olfactory marker protein [OMP] label). OSN density was calculated on single-plane images by counting the number of clearly OMP-positive somas (OMP label surrounding NucRed^+^ nucleus), divided by the length of the OE in that image, ×100 for comparative purposes (Kikuta et al., 2015; Cheetham et al., 2016). To quantify cell apoptosis, expressed as cells/mm for comparative purposes, the number of caspase-3-positive cells was measured in a whole z stack, and then divided by the total length of the OE in the stack (OE length × *n* of *z* steps) (Kikuta et al., 2015).

For activity marker genes and TH expression in the OB, images were taken with a 1× zoom (0.415 µm/pixel), 512 × 512 pixels, and in *z* stacks with 1 µm steps, with identical laser power and digital gain/offset settings within each set. In all animals, images were sampled from the rostral third, middle third, and caudal third of the OB. To avoid selection biases, all cells present in the stack and positive for the identifying marker (TH or SMI-32) were measured. DA cell density was calculated for each image by dividing the number of analyzed TH-positive cells by the volume of the glomerular layer (*z* depth × glomerular layer area, drawn and measured in a maximum intensity projection of the TH channel). SMI-32-positive mitral/tufted cells (M/TCs) were selected by position in the mitral layer; SMI-32-positive external tufted cells (ETCs) were included in the analysis only if their soma bordered with both the glomerular layer and external plexiform layer. TH-positive DA cells were included in the analysis only if their soma was in or bordering with the glomerular layer. Soma area was measured at the single plane including the cell's maximum diameter, by drawing an ROI with the free-hand drawing tool. Within each co-embedded set, the staining intensity of each ROI (expressed as mean gray value) was normalized to the mean value of staining intensity across all measured cells in the Ctrl slice. For analyses of within- versus between-mouse staining variability, the mean gray value of each M/TC pS6 ROI was normalized to the mean value across all measured cells in the right-OB slice from Mouse 1. Mean normalized intensities were then calculated for each slice, and absolute differences in these mean intensities were taken between the left- and right-bulb slices from Mouse 1 (for intra-animal variation), and between the slice from the Mouse 2 and both left- and right-bulb slices from Mouse 1. These two separate between-mouse differences were averaged to give an overall estimate of interanimal variation, which was compared with intra-animal variation on a slide-by-slide basis in a paired design. Staining intensity in AIS-positive DA cells (*i.e*., AnkG^+^/TH^+^) was normalized within each slide (rostral/middle/caudal) of each set, to the average TH or cFos staining of the overall DA cell population in the Ctrl slice.

For AIS identification, images were taken with 3× zoom, 512 × 512 pixels (0.138 µm/pixel) and in *z* stacks with 0.45 µm steps. While in all glutamatergic neurons only one extensive AnkG-positive region could be found on the proximal part of a process originating directly from the soma, DA cells' AISs were found either on processes originating directly from the soma (“soma-origin”) or on a process that did not originate directly from the soma (“dendrite-origin”). Moreover, as previously reported in the literature (Meyer and Wahle, 1988; Kosaka et al., 2008), a minority of DA cells was found to carry multiple AISs (10% of all imaged cells); these were excluded from further analysis. In all cells carrying a single AIS, its distance from soma and length were measured in Fiji/ImageJ using the View5D plugin, which allows for 3D manual tracing of cell processes. Laser power and gain settings were adjusted to prevent signal saturation in the AIS label AnkG; cellular marker TH or SMI-32 signal was usually saturated to enable clear delineation of the axon. The AIS distance from soma was calculated as the neurite path distance between the start of the AIS (the proximal point where AnkG staining became clearly identifiable) and the intersection of its primary parent process (usually the axon, but in the case of dendrite-origin axons the axon-bearing primary dendrite) with the border of the soma. AIS length was calculated by following AnkG staining along the course of the axon from the AIS start position to the point where AnkG staining was no longer clearly identifiable. To confirm the reliability of this manual tracing method, a subset of 50 AISs was analyzed twice by EG, blindly and with 2 weeks' interanalysis interval. Measurements of both distance from soma and length were highly consistent between the two analysis sessions (AIS distance from soma: difference mean ± SEM, 0.006 ± 0.097 µm, *r*^2^ = 0.75; AIS length: difference 0.139 ± 0.195 µm, *r*^2^ = 0.95). Relative AnkG mean staining intensity in axon-bearing DA cells was measured by drawing a freehand line along the AIS profile at the single *z* plane that contained the longest segment of the AIS. This process was repeated for all other AISs present in the same image stack, regardless of cellular origin (*i.e*., from ETCs and other interneurons), and the average staining intensity per stack was used for normalization.

##### Acute-slice electrophysiology

P21-35 C57BL/6 or DAT^IRES^*^cre^* x Ai9 (DAT-tdTomato) mice were decapitated under isoflurane anesthesia, and the OB was removed and transferred into ice-cold slicing medium containing the following (in mm): 240 sucrose, 5 KCl, 1.25 NaH_2_PO_4_, 2 MgSO_4_, 1 CaCl_2_, 26 NaHCO_3_, and 10 D-glucose, bubbled with 95% O_2_ and 5% CO_2_. Horizontal slices (300 µm thick) of the OB were cut using a vibratome (VT1000S, Leica Microsystems) and maintained in ACSF containing the following (in mm): 124 NaCl, 5 KCl, 1.25 NaH_2_PO_4_, 2 MgSO_4_, 2 CaCl_2_, 26 NaHCO_3_, and 20 D-glucose, bubbled with 95% O_2_ and 5% CO_2_ for >1 h before experiments began.

Whole-cell patch-clamp recordings were performed using a Multiclamp 700B amplifier (Molecular Devices) at physiologically-relevant temperature (32°C-34°C) with an inline heater (TC-344B, Warner Instruments). Signals were digitized (Digidata 1550, Molecular Devices) and Bessel-filtered at 3 kHz (membrane test pulses) or 10 kHz (all other protocols). Test recordings in DAT-tdTomato neurons (*n* = 3; data not shown) confirmed that varying the Bessel filter between 2 and 30 kHz had no impact on fundamental waveform features around AP onset; filtering at 10 kHz was therefore not a limiting factor in identifying cell subtypes based on their spike shape (see below). Recordings were excluded if series or input resistances (assessed by −10 mV voltage steps following each test pulse, acquisition rate 20 kHz) were, respectively, >30 mΩ or <100 mΩ for DA neurons, >30 mΩ or <30 mΩ for ETCs, >20 mΩ or <40 mΩ for M/TCs, or if they varied by >20% over the course of the experiment. Fast capacitance was compensated in the on-cell configuration and slow capacitance was compensated after rupture. Cell capacitance was calculated by measuring the area under the curve of the transient capacitive current elicited by a −10 mV voltage step. Resting membrane potential (*V_m_*) was assessed immediately after break-in by reading the voltage value in the absence of current injection (I = 0 configuration). Recording electrodes (GT100T-10, Harvard Apparatus) were pulled with a vertical puller (PC-10, Narishige) and filled with an intracellular solution containing the following (in mm): 124 K-gluconate, 9 KCl, 10 KOH, 4 NaCl, 10 HEPES, 28.5 sucrose, 4 Na_2_ATP, 0.4 Na_3_GTP (pH 7.25-7.35; 290 MOsm) and Alexa-488 (1:150). Cells were visualized using an upright microscope (Axioskop Eclipse FN1 Nikon) equipped with a 40× water immersion objective; and for DA cell identification, tdT fluorescence was revealed by LED (CoolLED pE-100) excitation with appropriate excitation and emission filters (ET575/50m, CAIRN Research). M/TCs were identified based on location in the mitral cell layer and large somas. ETCs were identified based on the following: (1) location in the lower glomerular layer/upper external plexiform layer; (2) large and balloon-shaped soma and, often, visible large apical dendrite; (3) characteristic spontaneous burst firing when unclamped; (4) an relatively depolarized resting membrane potential of ∼−55 mV; and (5) distinct depolarizing sag potential when injected with prolonged negative current steps in current-clamp mode (S. Liu and Shipley, 2008; Liu et al., 2013).

In current-clamp mode, evoked spikes were measured with *V*_hold_ set to −60 ± 3 mV for M/TCs and DA cells, and to −55 ± 3 mV for ETCs. For AP waveform measures, we injected 10-ms-duration current steps from 0 pA of increasing amplitude (Δ5/20 pA) until we reached the current threshold at which the neuron reliably fired an AP (*V*_m_ > 0 mV; acquisition rate 200 kHz). For multiple spiking measures, we injected 500-ms-duration current steps from 0 pA of increasing amplitude (Δ2/10 pA) until the neuron passed its maximum firing frequency (acquisition rate 50 kHz). Exported traces were analyzed using either ClampFit (pClamp10, Molecular Devices) or custom-written routines in MATLAB (The MathWorks). Before differentiation for dV/dt and associated phase plane plot analyses, recordings at high temporal resolution (5 µs sample interval) were smoothed using a 20 point (100 μs) sliding filter. Voltage threshold was taken as the potential at which dV/dt first passed 10 V/s. Onset rapidness was taken from the slope of a linear fit to the phase plane plot at voltage threshold. Spike width was measured at the midpoint between voltage threshold and maximum voltage. Rheobase and afterhyperpolarization (AHP) values were both measured from responses to 500 ms current injection, the latter from the local voltage minimum after the first spike fired at rheobase. Input-output curves were constructed by simply counting the number of spikes fired at each level of injected current.

For DA cells, monophasic versus biphasic phase plane plots were visually determined by EG and MSG. We classified completely monotonic plots with continually increasing rate of rise as monophasic, and any plots showing a clear inflection in rate of rise over the initial rising phase as biphasic. Any discrepancies in classification were resolved by mutual agreement. We also corroborated our subjective classification using a quantitative measure of spike onset sharpness: the ratio of errors produced by linear and exponential fits to the peri-threshold portion of the phase plane plot (Volgushev et al., 2008; Baranauskas et al., 2010). Fit error ratios were calculated with a custom MATLAB script written by Maxim Volgushev, using variable initial portions of the phase plane plot between voltage threshold and 40% of maximum dV/dt (Baranauskas et al., 2010) for single spikes fired in response to 10 ms current injection at current threshold and up to three subsequent suprathreshold sweeps (Galliano et al., 2018). In M/TC recordings, as expected for large projection neurons with a prominent AIS (Volgushev et al., 2008), these fit error ratios were consistently high (mean ± SEM, 5.45 ± 0.58 at 20% maximum dV/dt, *n* = 35), reflecting their markedly sharp spike onset, even in the absence of a clearly biphasic phase plane plot profile. In DA cells, we used strict, established (Baranauskas et al., 2010), but noninclusive criteria for “steep” (≈ biphasic; maximum fit error ratio >3) versus “smooth” (≈ monophasic; maximum fit error ratio <1) spike onset. This enabled us to objectively classify phase plane plot shape in a smaller subset (*n* = 28 of 48, = 58%) of our recorded DAT-tdTomato neurons. This quantitatively characterized subset included just 3 cells (11%), which were classified differently by our subjective versus objective criteria. Importantly, excluding these differentially classified cells from our analyses made no difference to any of our results in terms of significance.

##### Statistical analysis

Statistical analysis was conducted using Prism (GraphPad), SPSS (IBM), or MATLAB (The MathWorks). Sample distributions were assessed for normality with the D'Agostino and Pearson omnibus test, and parametric or nonparametric tests conducted accordingly. α values were set to 0.05, and all comparisons were two-tailed. For multilevel analyses, non-normal distributions were rendered normal by logarithmic transform. These parameters were then analyzed using linear mixed models (SPSS) with mouse or set as the subject variable (Aarts et al., 2014).

## Results

### Brief unilateral naris occlusion leaves the OE undamaged

Olfactory sensory deprivation in mice can be achieved surgically by cauterization of one naris, or mechanically by insertion of a custom-made and removable nasal plug (Coppola, 2012). Traditionally, both methods have been used for prolonged periods (weeks, months at a time), and are accompanied by pronounced and widespread changes in OB architecture, including overall OB size. This scenario is potentially pathological and does not reflect the most common deprivation that this sensory system has to deal with: a nasal blockage lasting <5 days (Fokkens et al., 2012).

**Figure 1.**
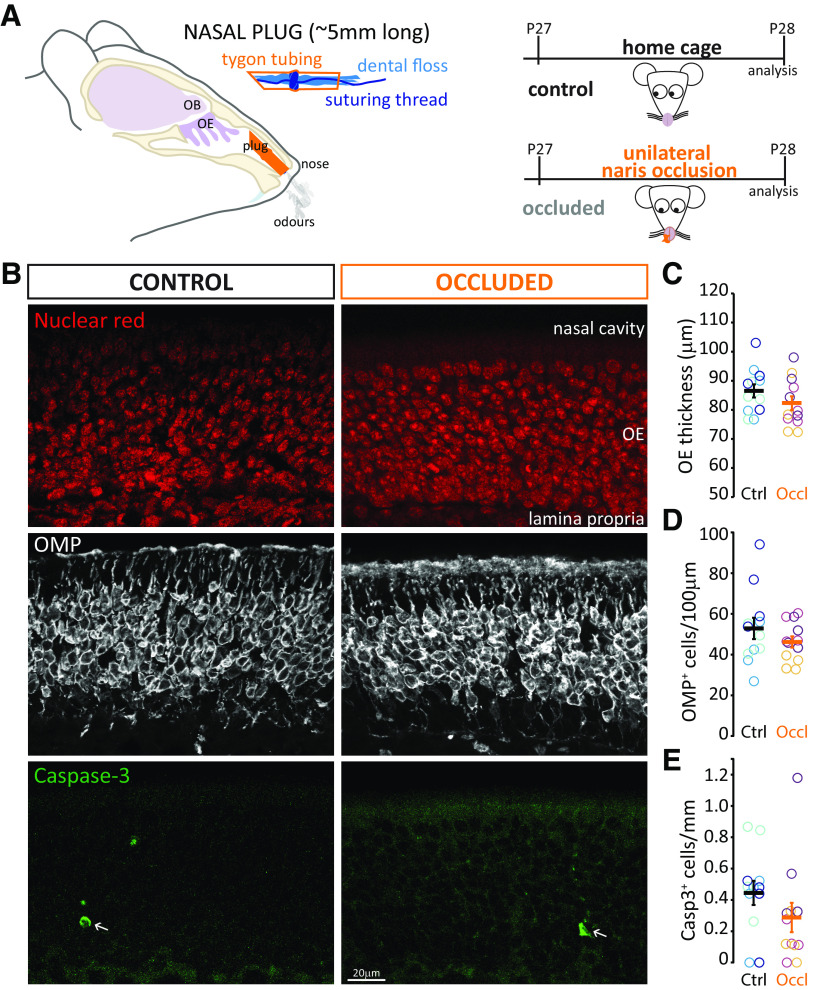
Brief unilateral naris occlusion does not damage the OE. ***A***, Left, Schematic representation of the custom-made plug (orange) blocking air flow in the mouse nasal cavity without contacting the OE. Right, Timeline of sensory manipulation. ***B***, Example images of olfactory epithelia in Ctrl and Occl mice. Arrow indicates rare caspase-3-positive cells. ***C***, Thickness of the OE in Ctrl and Occl mice. ***D***, Density of OMP-positive cells in Ctrl and Occl mice. ***E***, Density of caspase-3-positive cells in Ctrl and Occl mice. ***C–E***, Empty circles represent individual sample regions. Different colors represent different mice. Thick line indicates mean ± SEM.

In order to induce activity-dependent plasticity within a more naturally relevant timeframe, we used the custom-made plug method (Cummings and Brunjes, 1997) but left the plug in place for just one day (Fig. 1*A*). This 24 h duration is longer than the natural subcircadian cycles of relative air flow alternation between the nostrils (Bojsen-Moller and Fahrenkrug, 1971; Kahana-Zweig et al., 2016) but is well within the range of common infection-induced nasal blockade (Fokkens et al., 2012). We also chose it because we knew one day of activity manipulation was sufficient to produce multiple forms of plasticity in cultured OB neurons (Chand et al., 2015). Because of concerns regarding abnormal airflow through the remaining open nostril in unilaterally occluded animals (Coppola, 2012; Kass et al., 2013; Wu et al., 2017), we did not compare open and occluded hemispheres within the same experimental animals. Instead, juvenile (P27) WT mice were either left unperturbed (Fig. 1*A*; Ctrl group, black) or had one nostril plugged for 24 h (Occl group, orange), before being perfused and processed for immunohistochemistry.

To confirm the expected lack of peripheral pathology with this approach (Kikuta et al., 2015; Cheetham et al., 2016), we assessed the impact of plug insertion on the OE (Fig. 1*B*). We found no difference between Ctrl and 24 h Occl groups in overall OE thickness (Fig. 1*C*; Ctrl mean ± SEM, 86.51 ± 2.26 µm, *n* = 12 sample regions, *N* = 3 mice; Occl 82.26 ± 2.40 µm *n* = 12 sample regions, *N* = 3 mice; mixed-model ANOVA nested on mouse, effect of treatment, *F*_(1,24)_ = 1.81, *p* = 0.19). Similarly, the density of mature OSNs (identified by immunolabel for OMP) did not differ between Ctrl and Occl mice (Fig. 1*D*; Ctrl mean ± SEM, 52.84 ± 5.24 cells/100 µm, *n* = 12 sample regions, *N* = 3 mice; Occl 46.20 ± 2.78 cells/100 µm, *n* = 12 sample regions, *N* = 3 mice; mixed-model ANOVA nested on mouse, effect of treatment, *F*_(1,6)_ = 0.584, *p* = 0.47), nor did the density of apoptotic cells positive for activated caspase-3 (Fig. 1*E*; Ctrl mean ± SEM, 0.39 ± 0.084 cells/mm, *n* = 12 sample regions, *N* = 3 mice; Occl 0.29 ± 0.094 cells/mm, *n* = 12 sample regions, *N* = 3 mice; mixed-model ANOVA nested on mouse, effect of treatment, *F*_(1,6)_ = 0.423, *p* = 0.54). Overall, these data suggest that brief olfactory deprivation conducted with a custom-made plug has no impact on the overall structure and health of the OE.

### Brief unilateral naris occlusion alters the activity of inhibitory and excitatory bulbar neurons

Given that our chosen sensory manipulation is well within naturally experienced timeframes (Fokkens et al., 2012) and does not overtly damage the peripheral sense organ, we next checked that it was effective in reducing ongoing activity levels in downstream OB neurons.

**Figure 2.**
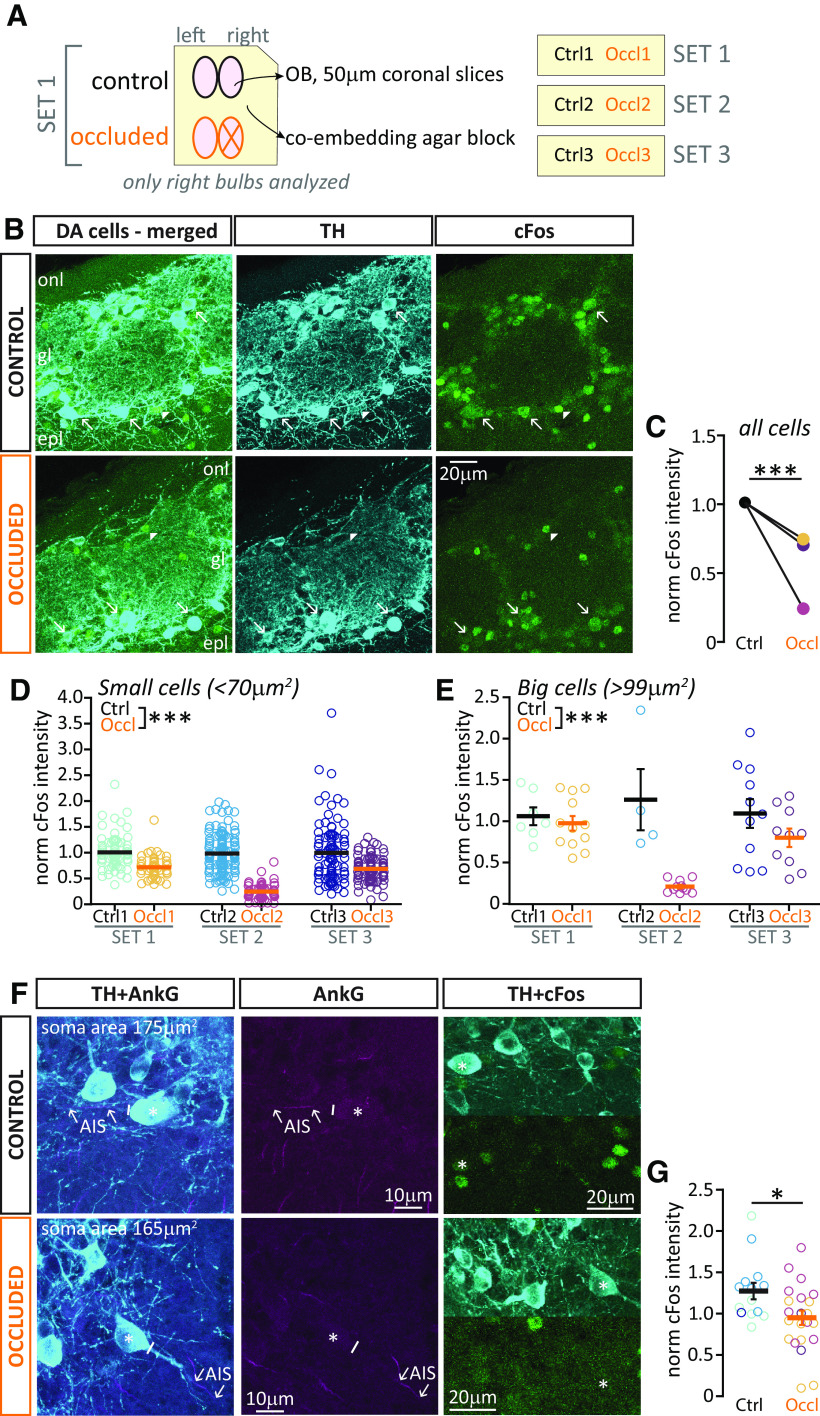
Brief unilateral naris occlusion decreases activity levels in both major subtypes of OB DA neurons. ***A***, Schematic representation of the experimental design: coronal OB slices from 1 Ctrl and 1 Occl (X) mouse were co-embedded in an agarose block (“set”) and processed and analyzed together (see Materials and Methods). ***B***, Example maximum intensity projection image of DA neurons visualized via anti-TH staining, and label for the activity early gene cFos, in Ctrl and Occl mice. The brightness of the TH channel has been adjusted independently in these Ctrl and Occl example images (dimmed and enhanced, respectively) to make DA cell identity clear; the cFos channels have not been altered. onl, Olfactory nerve layer; gl, glomerular layer; epl, external plexiform layer. Arrows indicate TH^+^/cFos^+^ cells. Arrowheads indicate TH^–^/cFos^+^ cells. ***C***, Mean normalized cFos intensity in TH^+^ cells of any soma size in Ctrl and Occl mice. ***D***, Normalized cFos intensity in TH^+^ cells with soma area < 70 µm^2^ (putative anaxonic DA cells), from 3 sets of Ctrl and Occl mice. ***E***, Normalized cFos intensity in TH^+^ cells with soma size > 99 µm^2^ (putative axon-bearing DA cells), from 3 sets of Ctrl and Occl mice. ***F***, Example images of cFos expression in TH^+^ cells with an identified AnkG^+^ AIS (arrows). Solid line indicates the emergence of the axonal process from the soma (asterisk). Note the different levels of cFos signal and background in the two example images, which were taken from the same co-embedded set but from different slices. ***G***, Normalized cFos intensity in AnkG^+^/TH^+^ cells in Ctrl and Occl mice. ***D***, ***E***, ***G***, Empty circles represent individual cells. Different colors indicate different mice. Thick lines indicate mean ± SEM. *p < 0.05. ***p < 0.0001.

We processed the OBs of Ctrl and Occl mice to quantify the expression of activity markers with immunohistochemistry. To control for differences in antibody exposure, we co-embedded slices from Ctrl and Occl mice in agarose blocks (“sets,” Fig. 2*A*) for consistent histological processing, and normalized activity marker intensity within each set (see Materials and Methods). We confirmed that this approach was effective in reducing interanimal staining variability by analyzing a separate group of co-embedded sets, which each contained slices from both the left and right OB of one unperturbed Ctrl mouse (allowing comparison of within-mouse variation between the two bulbs), plus an OB slice from a second unperturbed Ctrl mouse (allowing comparison of between-mouse variation; see Materials and Methods). In these analyses of tissue that all came from the same treatment group, we found that within-mouse absolute differences in mean staining intensity were not significantly different from between-mouse differences (paired *t* test, *t*_(8)_ = 1.02, *n* = 9 slides, *p* = 0.34), suggesting that our approach of slice co-embedding and standardized histological processing was sufficiently effective to reduce inter-animal variation down to the level of intra-animal variation.

We first analyzed the expression of the immediate early gene cFos (Barnes et al., 2015) in DA inhibitory neurons (DA cells, identified via TH immunoreactivity; Fig. 2*B*). DA cells in Occl bulbs displayed markedly and consistently lower spontaneous activity-related cFos levels than their co-embedded Ctrl counterparts, and this effect was highly significant in multilevel statistical analyses that account for inter-set variation (Fig. 2*B*; Ctrl mean ± SEM, 1 ± 0.02, *n* = 369 cells, *N* = 3 sets; Occl 0.56 ± 0.02, *n* = 301 cells, *N* = 3 sets; mixed-model ANOVA nested on set, effect of treatment *F*_(1,667)_ = 233, *p* < 0.0001).

Previous work from ourselves and others has found that bulbar DA neurons are a heterogeneous population (Chand et al., 2015; Galliano et al., 2018; Korshunov et al., 2020; Kosaka et al., 2020). Two non-overlapping subtypes can be identified by a spectrum of different morphological and functional characteristics, as well as by a binary classifier: the presence or absence of an axon and its key component, the AIS (Chand et al., 2015; Galliano et al., 2018). So, does brief unilateral naris occlusion downregulate activity in both axon-bearing and anaxonic DA subtypes? Soma size is a readily obtainable proxy indicator for DA subtypes: anaxonic DA cells are usually small, whereas axon-bearing DA cells tend to have very large somas. Using previously defined lower (<70 µm^2^) and upper (>99 µm^2^) bounds of the OB DA soma size distribution (Galliano et al., 2018), we found that both small/putative anaxonic DA cells and large/putative axon-bearing DA cells from Occl mice displayed reduced cFos staining relative to their co-embedded Ctrl counterparts. Although the smaller sample size of the much rarer large DA cells accentuated variability across staining sets here, this effect was highly significant for both cell types in analyses that specifically account for that variation (small cells: Ctrl mean ± SEM, 0.99 ± 0.03, *n* = 298 cells from *N* = 3 sets; Occl 0.56 ± 0.02, *n* = 192 cells, *N* = 3 sets; mixed-model ANOVA nested on mouse, effect of treatment *F*_(1,489)_ = 166, *p* < 0.0001; big cells: Ctrl mean ± SEM, 1.11 ± 0.11, *n* = 22 cells from *N* = 3 sets; Occl 0.67 ±0.07, *n* = 33 cells, *N* = 3 sets; mixed-model ANOVA nested on mouse, effect of treatment, *F*_(1,53)_ = 11.91, *p* < 0.0001). Finally, to further confirm these results in DA cells which definitively belonged to the axon-bearing subtype, we costained a subset of tissue with the AIS marker AnkG and measured cFos levels in AnkG^+^/TH^+^ DA cells (Fig. 2*F*; see Materials and Methods). Once more, we found significantly dimmer cFos fluorescence in Occl cells (Fig. 2*G*; Ctrl mean ± SEM, 1.28 ± 0.10, *n* = 14 cells, Occl 0.95 ± 0.09, *n* = 22 cells, Mann–Whitney, *U* = 82, *p* = 0.02).

**Figure 3.**
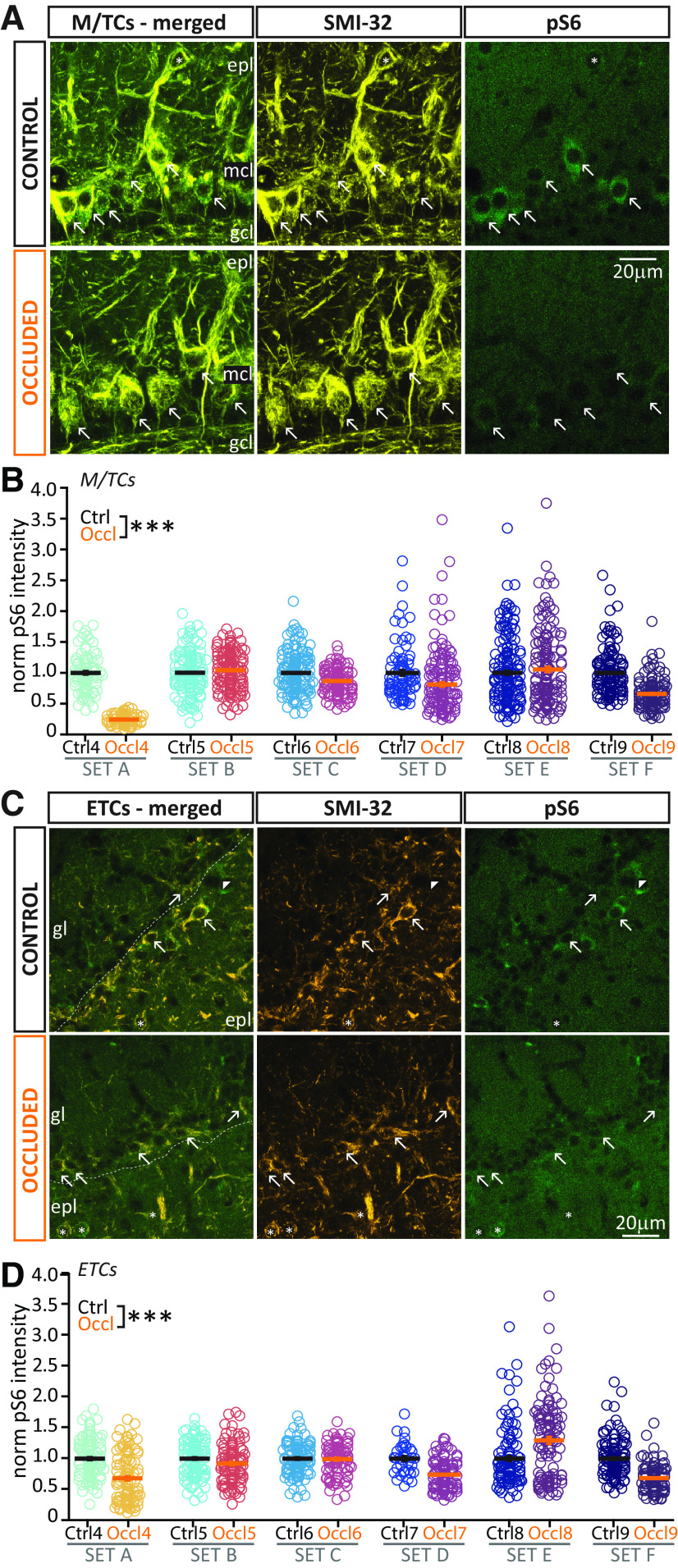
Brief unilateral naris occlusion decreases activity levels in bulbar excitatory neurons. ***A***, ***C***, Example maximum intensity projection images of bulbar M/TCs (***A***) or ETCs (***C***) visualized via SMI-32 staining, and the activity marker pS6. epl, External plexiform layer; mcl, mitral cell layer; gcl, granule cell layer; gl, glomerular layer. Arrows indicate pS6^+^ M/TCs (***A***) or ETCs (***C***). SMI-32^+^ cells located in the epl (asterisks) were not analyzed. Experimental design as in Figure 2*A*. ***B***, ***D***, Normalized pS6 intensity in M/TCs (***B***) or ETCs (***D***) from 6 sets of Ctrl and Occl mice. Empty circles represent individual cells. Different colors represent different mice. Thick line indicates mean ± SEM. ***p < 0.0001.

This effect of naris occlusion on activity levels was more variable, but nevertheless also present overall in bulbar glutamatergic neurons. These belong to two main classes defined by location and axonal projections: M/TCs and ETCs. M/TCs (Fig. 3*A*), whose soma sits in the mitral cell layer, are the bulbar network's principal neurons; they extend their apical dendrites to the glomerular layer where they receive direct and indirect inputs from OSNs, and send their axons to higher olfactory areas, including piriform cortex (Imai, 2014). ETCs (Fig. 3*C*) are glutamatergic interneurons located in the glomerular layer, where they provide local dendrodendritic amplification of sensory inputs (Najac et al., 2011; Gire et al., 2012). ETC axons do not leave the OB, but target deep-layer networks beneath sister glomeruli in the opposite hemi-bulb (Lodovichi et al., 2003; Cummings and Belluscio, 2010). To identify both classes of excitatory neurons, we labeled bulbar slices with the neurofilament marker protein H, clone SMI-32 (Table 1).

We co-stained with antibodies against another activity marker, phospho-S6 ribosomal protein (pS6) (Knight et al., 2012), which in bulbar glutamatergic cells gives higher intensity and consistency of staining than cFos (Fig. 3*A*,*C*). Using a co-embedding approach to allow comparisons of relative staining intensity across slices (Fig. 2*A*; see Materials and Methods), we found that, in both M/TCs and ETCs from Occl slices, the relative intensity levels of pS6 were markedly variable across staining sets (see set-by-set comparisons in Fig. 3*B*,*D*). This may be because of cell- and/or marker-type differences in activity changes occurring during brief sensory deprivation. Mouse-to-mouse differences in the efficacy of naris block may also play a role here, although the more consistent effects of occlusion on cFos staining in DA cells (Fig. 2*C*) (see also Byrne et al., 2020) suggest that this is not a strong contributing factor. To account for the considerable set-to-set variability in our pS6 data, we used multilevel statistical analyses with our cell-by-cell data nested by co-embedded set (Aarts et al., 2014), finding that pS6 intensity was significantly decreased overall in both cell types in Occl bulbs compared with co-embedded Ctrls (M/TC Ctrl mean ± SEM, 1.00 ± 0.013, *n* = 858 cells; Occl 0.80 ± 0.015, *n* = 930 cells, *N* = 6 sets; mixed-model ANOVA nested on set, effect of treatment, *F*_(1,1783)_ = 94, *p* < 0.0001; Fig. 3*B*; ETC Ctrl 1.00 ± 0.012, *n* = 642 cells; Occl 0.89 ± 0.018, *n* = 624 cells, *N* = 6 sets; mixed-model ANOVA nested on set, effect of treatment, *F*_(1,1264)_ = 22 *p* < 0.0001; Fig. 3*D*).

In summary, despite some mouse-to-mouse variability, which is more marked for excitatory neurons, short-duration naris occlusion comparable to the sensory deprivation produced by a mild common cold (Fokkens et al., 2012) is effective overall in reducing activity levels in multiple OB cell types.

### Lack of structural and intrinsic activity-dependent plasticity in excitatory neurons

Previous *in vitro* work from our laboratory has demonstrated that both GABAergic and GABA-negative neurons in bulbar dissociated cultures respond to 24 h manipulations of neuronal activity by modulating the length and/or position of their AIS (Chand et al., 2015). This finding raised a number of questions, namely, (1) whether AIS plasticity also occurs *in vivo* in response to a sensory manipulation of similar duration; (2) if so, in which cell types; and, finally (3) whether structural plasticity at the AIS is accompanied by functional plasticity of the neurons' intrinsic excitability.

In multiple cell types after 24 h naris occlusion, we performed *ex vivo* immunohistochemistry to quantify AIS position and length, and whole-cell patch-clamp recording in acute slices to assess neurons' passive and active electrophysiological properties.

**Figure 4.**
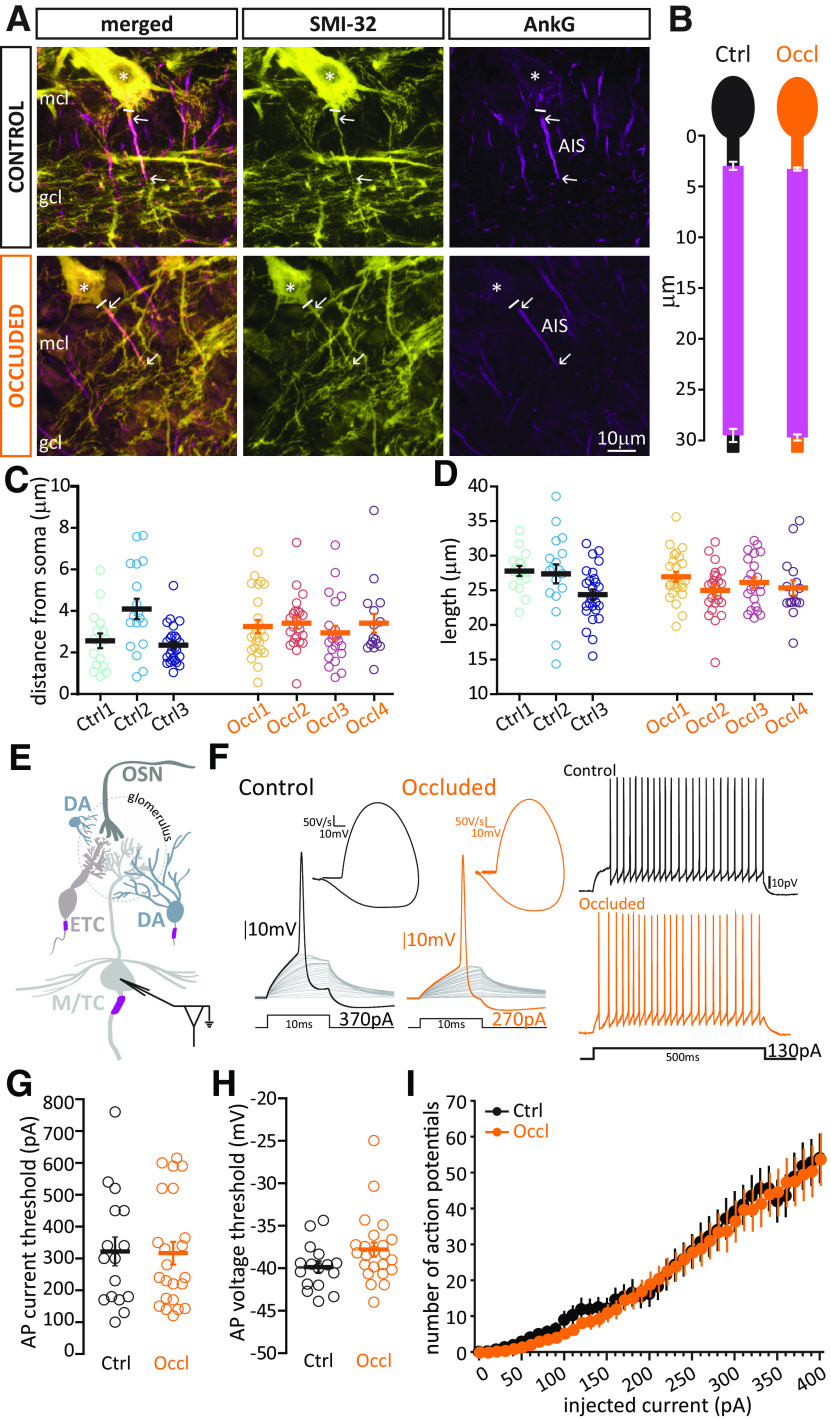
Brief unilateral naris occlusion fails to induce structural plasticity at the AIS or plasticity of intrinsic excitability in M/TCs. ***A***, Example average intensity projection image of bulbar M/TCs visualized via SMI-32 staining and the AIS marker AnkG in Ctrl and Occl mice. mcl, Mitral cell layer; gcl, granule cell layer. Solid line indicates the emergence of the axonal process from the soma (asterisk). Arrows indicate AIS start and end positions. ***B***, Mean ± SEM AIS start and end position for each group. ***C***, AIS distance from soma in M/TCs from Ctrl and Occl mice. ***D***, AIS length in M/TCs from Ctrl and Occl mice. ***C***, ***D***, Empty circles represent individual cells. Different colors represent different mice. Thick line indicates mean ± SEM. ***E***, Diagram of whole-cell recordings from M/TCs. ***F***, Left, Example current-clamp traces of single APs fired to threshold 10 ms somatic current injection by Ctrl and Occl M/TCs, and their associated phase plane plots. Right, Example current-clamp traces of multiple APs fired in response to a 130 pA/500 ms somatic current injection in Ctrl and Occl cells. ***G***, Single AP current threshold in Ctrl and Occl M/T cells. ***H***, Single AP voltage threshold in Ctrl and Occl M/TCs. ***G***, ***H***, Empty circles represent individual cells. Thick lines indicate mean ± SEM. ***I***, Input-output curve of 500 ms-duration current injection magnitude versus mean ± SEM spike number for each group.

In fixed slices of juvenile C57BL/6 mice, we identified M/TCs by staining the neurofilament protein H, clone SMI-32 (Ashwell, 2006). AISs were identified with staining against AnkG (Fig. 4*A*), and measured in 3D (see Materials and Methods). M/TCs all have a prominent and reliably oriented axon, which arises directly from the soma and projects toward the granule cell layer of the OB. Their AnkG-positive AISs tend to be ∼25 µm in length and proximally located (Lorincz and Nusser, 2008).

We found no difference in AIS distance from the soma (Ctrl mean ± SEM, 2.92 ± 0.21 µm, *n* = 61 cells, *N* = 3 mice; Occl, 3.25 ± 0.15 µm, *n* = 87 cells, *N* = 4 mice; mixed-model ANOVA of log-transformed AIS distance nested on mouse, effect of treatment, *F*_(1,6)_ = 1.24, *p* = 0.31), nor in AIS length (Ctrl, mean ± SEM, 26.17 ± 0.58 mm; Occl, 25.91 ± 0.40 mm; mixed-model ANOVA nested on mouse, effect of treatment *F*_(1,8)_ = 0.24, *p* = 0.64) between Ctrl and Occl M/TCs (Fig. 4*B-D*). This lack of structural AIS plasticity was mirrored by an equal absence of plastic changes in M/TCs' intrinsic excitability. When probed with short current injections (10 ms, Fig. 4*F*, left), Ctrl and Occl M/TCs fired an AP at similar thresholds, both in terms of injected current (Fig. 4*G*; Ctrl, mean ± SEM, 323 ± 45 pA, *n* = 16 cells; Occl, 317 ± 36 pA, *n* = 23 cells; unpaired *t* test, *t*_(37)_ = 0.098, *p* = 0.92) and somatic membrane voltage (Fig. 4*H*; Ctrl, mean ± SEM, −39.86 ± 0.67 mV, *n* = 16 cells; Occl, −37.80 ± 0.83 mV, *n* = 23 cells; Mann–Whitney test, *U* = 121, *p* = 0.07). These threshold single spikes in M/TCs were characterized by their markedly sharp onset, particularly clear in their spike phase-plane plots (Fig. 4*F*, left, insets), consistent with AP initiation away from the recording site, presumably in the AIS (see Materials and Methods) (Coombs et al., 1957; Jenerick, 1963; Khaliq et al., 2003; Bean, 2007; Kole et al., 2007; Shu et al., 2007; Foust et al., 2010; Bender and Trussell, 2012). When probed with longer 500 ms current injections to elicit repetitive AP firing (Fig. 4*F*, left), we again found no difference between the two groups (Fig. 4*I*; Ctrl *n* = 16 cells, Occl, *n* = 23 cells; mixed-model ANOVA, effect of treatment, *F*_(1,51)_ = 0.30, *p* = 0.59). Moreover, Ctrl and Occl M/TCs did not differ significantly in any other measured electrophysiological property, passive or active (Table 2).

**Table 2.** Intrinsic electrophysiological properties of M/TCs

	Ctrl (mean ± SEM, [*n*])	Occl (mean ± SEM, [*n*])	Test type, *p* value
**Passive properties**			
Membrane capacitance (pF)	66 ± 4, [24]	65 ± 4, [26]	*t*, 0.77
Resting membrane potential (mV)	−49.12 ± 1.318, [4]	−51.33 ± 1.535, [8]	*t*, 0.41
Input resistance (mΩ)	135 ± 19, [24]	108 ± 11, [26]	MW, 0.33
**Action potential properties**			
Threshold (pA)	323 ± 45, [16]	317 ± 36, [23]	*t*, 0.92
Threshold (mV)	−39.86 ± 0.67, [16]	−37.80 ± 0.83, [23]	MW, 0.07
Maximum voltage reached (mV)	29.29 ± 1.64, [16]	30.81 ± 1.21, [23]	*t*, 0.45
Peak amplitude (mV)	69.15 ± 1.38, [16]	68.61 ± 1.28, [23]	*t*, 0.78
Width at half-height (ms)	0.41 ± 0.02, [15]	0.45 ± 0.02, [23]	*t*, 0.15
Rate of rise (max dV/dt, mV*ms)	366 ± 16, [16]	346 ± 12, [23]	*t*, 0.32
Onset rapidness (1/ms)	32.68 ± 1.28, [16]	27.34 ± 2.05, [23]	*t*, 0.054
AHP (mV)	−54.12 ± 0.85, [22]	−53.88 ± 0.59, [24]	*t*, 0.82
AHP relative to threshold (mV)	16.24 ± 0.74, [22]	18.16 ± 0.80, [24]	*t*, 0.09
**Repetitive firing properties**			
Rheobase (pA)	108 ± 17.72, [22]	122.2 ± 16.06, [25]	*t*, 0.55
Maximum no. of APs	64.39 ± 6.33, [22]	58.75 ± 6.16, [25]	*t*, 0.53
First AP delay (ms)	392 ± 16, [22]	368 ± 18, [25]	*t*, 0.33
Interspike interval CV	0.48 ± 0.07, [22]	0.38 ± 0.05, [26]	MW, 0.44

Data are mean ± SEM values of passive, AP, and repetitive firing properties for Ctrl and Occl M/T cells. Statistical differences between groups were calculated with an unpaired *t* test for normally distributed data (*t*) or with a Mann–Whitney test for non-normally distributed data (MW).

**Figure 5.**
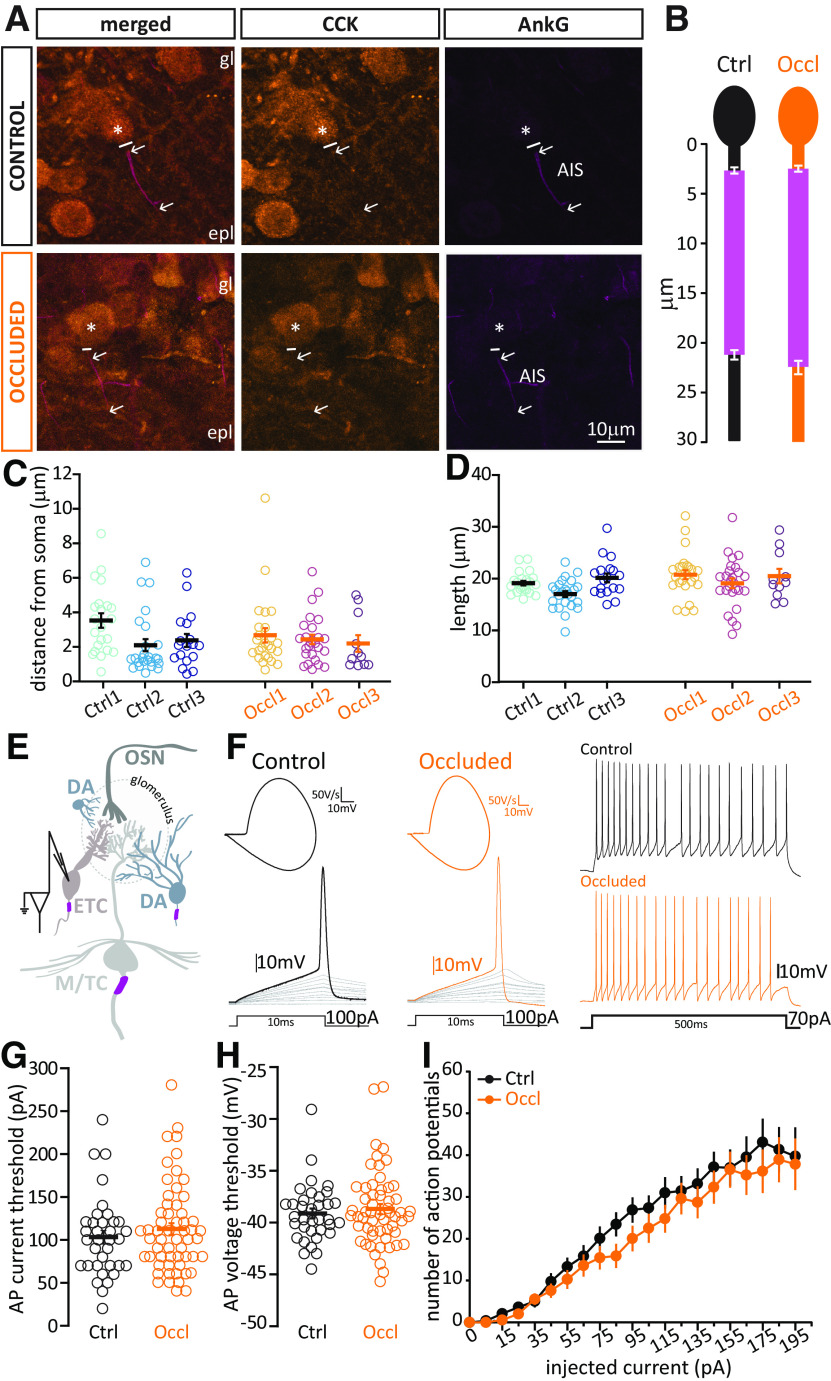
Brief unilateral naris occlusion fails to induce structural plasticity at the AIS or plasticity of intrinsic excitability in ETCs. ***A***, Example average intensity projection images of bulbar ETCs visualized via staining against CCK and the AIS marker AnkG in Ctrl and Occl mice. gl, Glomerular layer; epl, external plexiform layer. ***B–I***, All conventions are the same as in Figure 4.

Similarly, we also found no evidence for structural or intrinsic activity-dependent plasticity in ETCs. In these experiments, we visualized ETCs in fixed tissue by staining for CCK (Fig. 4*A*) (Liu and Shipley, 1994). We found that, as for M/TCs, ETC AISs are prominent AnkG-positive segments located quite proximally on a process originating directly from the soma. These AISs were equally distant from the soma (Fig. 5*C*; Ctrl mean ± SEM, 2.67 ± 0.23 µm, *n* = 65 cells, *N* = 3 mice; Occl, 2.496 ± 0.22 µm, *n* = 62 cells, *N* = 3 mice; mixed-model ANOVA of log-transformed AIS distance nested on mouse, effect of treatment, *F*_(1,6)_ = 0.018, *p* = 0.90) and equally long (Fig. 5*D*; Ctrl mean ± SEM, 18.52 ± 0.39 µm, *n* = 65 cells, *N* = 3 mice; Occl, 19.94 ± 0.59 µm, *n* = 62 cells, *N* = 3 mice; mixed-model ANOVA nested on mouse, effect of treatment, *F*_(1,6)_ = 2.31, *p* = 0.18) in Ctrl and Occl mice. Moreover, when probed electrophysiologically in acute slices (Fig. 5*F*,*G*; Table 3), ETCs from Ctrl and Occl mice fired sharp-onset single APs at similar thresholds (current threshold, Fig. 5*G*, Ctrl mean ± SEM, 103 ± 8 pA, *n* = 35 cells; Occl, 112 ± 7 pA, *n* = 57 cells, Mann–Whitney test, *U* = 913, *p* = 0.50; voltage threshold, Fig. 5*H*, Ctrl −39.10 ± 0.48 mV, *n* = 35 cells; Occl −38.56 ± 0.48 mV, *n* = 57 cells; Mann–Whitney test, *U* = 926, *p* = 0.57), and similarly modulated their repetitive firing in response to long current injections of increasing intensity (Fig. 5*I*; Ctrl *n* = 30 cells, Occl *n* = 45 cells; mixed-model ANOVA, effect of treatment, *F*_(1,96)_ = 1.80, *p* = 0.18).

**Table 3.** Intrinsic electrophysiological properties of ETCs

	Ctrl (mean ± SEM, [*n*])	Occl (mean ± SEM, [*n*])	Test type, *p* value
**Passive properties**			
Membrane capacitance (pF)	43.43 ± 2.13, [35]	41.92 ± 1.67, [57]	*t*, 0.58
Resting membrane potential (mV)	−57.33 ± 0.92, [36]	−56.58 ± 0.94, [64]	*t*, 0.60
Input resistance (mΩ)	271 ± 27, [35]	242 ± 17, [57]	MW, 0.29
**Action potential properties**			
Threshold (pA)	103 ± 8, [35]	112 ± 7, [57]	MW, 0.50
Threshold (mV)	−39.10 ± 0.48, [35]	−38.56 ± 0.48, [57]	MW, 0.57
Maximum voltage reached (mV)	22.08 ± 1.18, [35]	22.69 ± 0.69, [57]	*t*, 0.64
Peak amplitude (mV)	61.17 ± 1.14, [35]	61.26 ± 0.82, [57]	*t*, 0.94
Width at half-height (ms)	0.53 ± 0.02, [35]	0.52 ± 0.01, [57]	MW, 0.94
Rate of rise (max dV/dt, mV*ms)	205 ± 7, [35]	211 ± 4, [57]	*t*, 0.44
Onset rapidness (1/ms)	29.65 ± 0.85, [30]	30.18 ± 0.94, [37]	MW, 0.42
AHP (mV)	−52.04 ± 0.45, [23]	−52.30 ± 0.60, [38]	*t*, 0.76
AHP relative to threshold (mV)	15.41 ± 0.85, [23]	17.14 ± 0.58, [38]	*t*, 0.09
**Repetitive firing properties**			
Rheobase (pA)	41 ± 8 pA, [23]	45 ± 7 pA, [38]	MW, 0.73
Maximum no. of APs	61 ± 4, [30]	56 ± 3, [45]	*t*, 0.34
First AP delay (ms)	187 ± 23, [23]	171 ± 22, [38]	*t*, 0.63
Interspike interval CV	0.33 ± 0.04, [30]	0.38 ± 0.04, [45]	MW, 0.65

Data are mean ± SEM values of passive, AP, and repetitive firing properties for Ctrl and Occl ET cells. Statistical differences between groups were calculated with an unpaired *t* test for normally distributed data (*t*) or with a Mann–Whitney test for non-normally distributed data (MW).

Together, these results confirm that, while both major classes of bulbar excitatory neurons experience an overall drop in activity after 24 h sensory deprivation (Fig. 2), they do not respond by altering the structural features of their AIS or their intrinsic physiological properties.

### Both inhibitory DA neuron subclasses downregulate their TH expression levels in response to brief naris occlusion

In other brain areas, inhibitory interneurons can act as first responders in the early phases of adaptation to changed incoming activity, plastically changing their overall structure and function to maintain circuit homeostasis (Knott et al., 2002; Hartmann et al., 2008; Yin and Yuan, 2014; Gainey and Feldman, 2017; Keck et al., 2017). Given the lack of plasticity in glutamatergic OB neurons following brief 24 h naris occlusion, we reasoned that plastic responses might therefore be more evident in OB inhibitory interneurons. Because of their well-documented plasticity *in vivo* and their ability to undergo activity-dependent AIS changes *in vitro* (Chand et al., 2015; Bonzano et al., 2016), we focused on the bulb's DA population to address this question.

Bulbar DA neurons are unique among other glomerular layer inhibitory neurons because of their well-described plasticity in neurotransmitter-synthesizing enzyme expression. Changes in sensory input, including those induced by unilateral naris occlusion, are known to produce alterations in TH expression at both the protein and mRNA levels (Nadi et al., 1981; Kosaka et al., 1987; Baker et al., 1993; Cummings and Brunjes, 1997). As with other forms of experience-dependent plasticity, these changes have been mostly investigated using long-duration manipulations. However, 2 days of deprivation were reported to induce a small, but significant, decrease in whole-bulb *Th* mRNA (Cho et al., 1996), whereas just one day of elevated activity was sufficient to increase TH immunofluorescence intensity or TH-GFP transgene expression, respectively, in dissociated and slice culture preparations (Akiba et al., 2007; Chand et al., 2015). We therefore set out to assess whether 24 h naris occlusion is sufficient to produce activity-dependent changes in TH expression *in vivo*, and if so whether these changes are observed in both axon-bearing and anaxonic OB DA subtypes.

**Figure 6.**
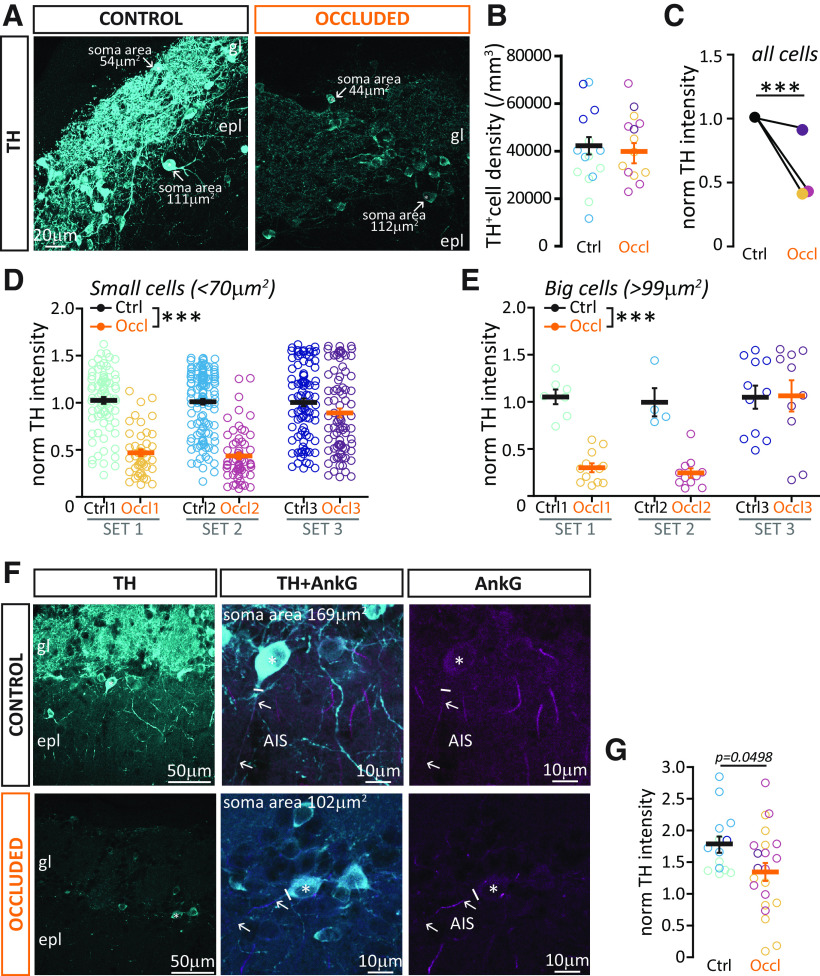
Brief unilateral naris occlusion decreases the expression of TH in both DA subtypes. ***A***, Example maximum intensity projection image of DA neurons visualized via TH immunolabel in Ctrl and Occl mice. The TH images here are unaltered, and acquired with identical settings. gl, Glomerular layer; epl, external plexiform layer. Arrows indicate TH^+^ DA cells representative of the two subtypes when defined by soma area. ***B***, Average glomerular layer density of TH^+^ cells (of any soma size) in Ctrl and Occl mice. Empty circles represent individual image stacks. Different colors represent different mice. Thick lines indicate mean ± SEM. ***C***, Mean normalized TH intensity in DA cells of any soma size, in 3 sets of Ctrl and Occl OBs. ***D***, Normalized TH intensity of DA cells with soma size < 70 µm^2^ (putative anaxonic cells), from 3 sets of Ctrl and Occl mice. ***E***, Normalized TH intensity of DA cells with soma size > 99 µm^2^ (putative axon-bearing DA cells), from 3 sets of Ctrl and Occl mice. ***D***, ***E***, Empty circles represent individual cells. Different colors represent different mice. Thick lines indicate mean ± SEM. ***p < 0.0001. ***F***, Example average intensity projection images of TH label in DA cells with an identified AnkG^+^ AIS (arrows). Solid line indicates the emergence of the axonal process from the soma (*). ***G***, Normalized TH intensity in AnkG^+^ DA cells in Ctrl and Occl mice. All conventions are the same as in ***D***.

In 3 sets of co-embedded Ctrl and Occl coronal bulbar slices (Fig. 2*A*) stained with an antibody against TH (Fig. 6*A*), we first confirmed that the overall density of labeled DA cells was unaffected by brief sensory deprivation (Fig. 6*B*; Ctrl mean ± SEM, 42,317 ± 3661 cells/mm^3^, *n* = 14 regions, *N* = 3 sets, Occl 39 993 ± 4243 cells/mm^3^, *n* = 15 regions, *N* = 3 sets; mixed-model ANOVA nested on set, effect of treatment, *F*_(1,25)_ = 0.39, *p* = 0.54). In the knowledge that we were labeling a similar number of TH^+^ cells in both groups, we then analyzed relative TH immunofluorescence levels in each set, normalizing the intensity of staining to average Ctrl values (see Materials and Methods). Given the interset variability noted in our cFos data (Fig. 2), it was unsurprising to also observe such variability in relative TH intensity levels. This was particularly evident in the smaller occlusion effect observed in set 3 here, and especially for the smaller sample of much rarer large neurons (Fig. 6*C-E*). We saw similar set-to-set variability in a separate analysis of TH immunofluorescence changes after 24 h occlusion (Byrne et al., 2020) but less variability in whole-bulb qPCR estimates of relative *Th* mRNA levels in that study. This suggests that set-to-set variation in relative TH staining intensity may be driven more by differences in locally imaged regions for immunofluorescence quantification, differences in staining between preparations, and/or more variable occlusion effects at the protein versus transcript level, rather than by mouse-to-mouse differences in the efficacy of naris block. Regardless of the causes of set-to-set variation, multilevel analyses that specifically take it into account revealed highly significant overall reductions in TH immunofluorescence levels in all DA cell groups. Significant changes were observed in all DA cells (Fig. 6*C*; Ctrl mean ± SEM, 1.00 ± 0.019, *n* = 369 cells, *N* = 3 sets; Occl 0.59 ± 0.023, *n* = 301 cells, *N* = 3 sets; mixed-model ANOVA nested on set, effect of treatment, *F*_(1,667)_ = 212, *p* < 0.0001), small putative anaxonic cells (Fig. 6*D*; Ctrl mean ± SEM, 1.01 ± 0.02, *n* = 298 cells, *N* = 3 sets; Occl 0.64 ± 0.03, *n* = 192 cells, *N* = 3 sets; mixed-model ANOVA nested on set, effect of treatment, *F*_(1,489)_ = 130, *p* < 0.0001), and large putative axon-bearing cells (Fig. 6*E*; Ctrl mean ± SEM, 1.01 ± 0.07, *n* = 22 cells, *N* = 3 sets; Occl 0.51 ± 0.08, *n* = 33 cells, *N* = 3 sets; mixed-model ANOVA nested on set, effect of treatment, *F*_(1,53)_ = 19, *p* < 0.0001). We further confirmed this latter phenotype in a smaller subset of DA cells with definitively identified AISs (Fig. 6*F*; normalized TH intensity; Ctrl mean ± SEM, 1.79 ± 0.13, *n* = 14, Occl 1.37 ± 0.14, *n* = 22, unpaired *t* test, *t*_(34)_ = 2.03; *p* = 0.0498). We also found significant positive correlations between normalized TH and normalized cFos (Fig. 2) intensities for all groups. These were stronger for Ctrl neurons (norm TH vs norm cFos, Ctrl small cells, Pearson *r* = 0.70, *n* = 298 cells, *p* < 0.0001; big cells, *r* = 0.89, *n* = 22, *p* < 0.0001; Occl small cells, *r* = 0.52, *n* = 192, *p* < 0.0001; big cells, *r* = 0.42, *n* = 33, *p* = 0.015), suggesting that the mechanisms leading to activity-dependent TH and cFos changes in individual OB DA neurons are only loosely coupled. Overall, given that alterations in OB TH levels are often used to confirm the effectiveness of olfactory sensory manipulations (Cockerham et al., 2009; Kass et al., 2013; Grier et al., 2016), these data supplement the immediate early gene analysis (Fig. 2) to show that 24 h naris occlusion strongly and reliably downregulates activity in both subclasses of OB DA interneurons. They also provide evidence for, to date, the fastest activity-dependent TH change observed in this cell class *in vivo* (see also Byrne et al., 2020).

### Anaxonic DA neurons do not modulate their intrinsic excitably following brief sensory deprivation

The vast majority of DA neurons are anaxonic cells (Galliano et al., 2018), which by locally releasing GABA and dopamine in the glomerular layer help to control the overall gain of OSN→M/TC transmission (McGann, 2013; Vaaga et al., 2017). Highly plastic, they retain the capability to regenerate throughout life (Lledo et al., 2006; De Marchis et al., 2007; Bonzano et al., 2016; Galliano et al., 2018). However, although they regulate their levels of TH expression in response to 24 h naris occlusion (Fig. 6), we found that the same manipulation did not change their intrinsic excitability.

**Figure 7.**
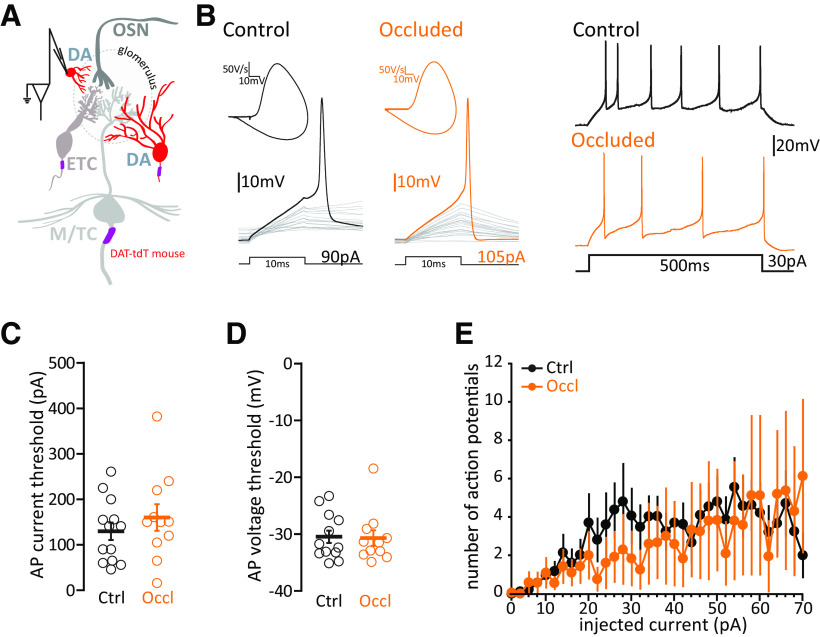
Brief unilateral naris occlusion does not alter the intrinsic excitability of monophasic/putative anaxonic DA cells. ***A***, Diagram of whole-cell recordings from small fluorescent cells in DAT-tdTomato mice. ***B–E***, All conventions are the same as in Figure 4*F–I*.

We performed whole-cell patch-clamp recordings in Ctrl and Occl DAT-tdTomato mice (Bäckman et al., 2006; Madisen et al., 2010). This transgenic labeling approach produces red fluorescent tdT-positive glomerular layer cells that are ∼75%-85% colabeled for TH (Fig. 7*A*) (Vaaga et al., 2017; Galliano et al., 2018; Byrne et al., 2020). The remaining tdT^+^/TH^–^ non–DA-labeled OB neurons in these mice are of the calretinin-expressing OB interneuron class and can be readily identified by their unique physiological properties (Pignatelli et al., 2005; Sanz Diez et al., 2019; Byrne et al., 2020), so these were excluded from our analyses. Anaxonic DA cells, which are overrepresented in DAT-tdTomato mice (Galliano et al., 2018), were functionally classified by assessing the nature of their AP phase plane plot of single spikes fired in response to 10 ms somatic current injection (Fig. 7*B*). A smooth, monophasic phase plane plot is indicative of AP initiation at the somatic recording site, and can be used as a proxy indicator of anaxonic morphology. In contrast, a distinctive biphasic phase plane plot waveform indicates that the AP initiated at a nonsomatic location, usually the AIS, and can be used as a proxy for axon-bearing identity (see Materials and Methods) (Coombs et al., 1957; Jenerick, 1963; Khaliq et al., 2003; Bean, 2007; Kole et al., 2007; Shu et al., 2007; Foust et al., 2010; Bender and Trussell, 2012; Chand et al., 2015; Galliano et al., 2018; Werginz et al., 2020). Indeed, we confirmed that monophasic, putative anaxonic cells had smaller soma sizes than putative axon-bearing neurons with biphasic phase plane plot signatures (see below; monophasic mean ± SEM, 56.36 ± 3.40 µm^2^, *n* = 25 cells; biphasic 89.44 ± 5.19 µm^2^, *n* = 21 cells; unpaired *t* test, *t*_(44)_ =5.49, *p* < 0.0001) (Chand et al., 2015; Galliano et al., 2018).

We found that, while sitting at a more depolarized resting membrane potential than their Ctrl counterparts, monophasic/putative-anaxonic DA cells from Occl mice showed no other significant differences in their passive membrane properties (Table 4). Measures of intrinsic excitability, importantly measured from identical baseline voltage, were indistinguishable between the two groups. Ctrl and Occl monophasic neurons fired single spikes at similar thresholds (current threshold, Fig. 7*D*; Ctrl mean ± SEM, 129.7 ± 19.2 pA, *n* = 13 cells; Occl, 160 ± 29.23 pA, *n* = 11 cells; unpaired *t* test, *t*_(22)_ = 0.89, *p* = 0.38; voltage threshold, Fig. 7*D*; Ctrl −30.47 ± 1.09 mV, *n* = 13 cells; Occl −30.70 ± 1.37 mV, *n* = 11 cells; Mann–Whitney test, *U* = 68, *p* = 0.86), and, when probed with longer current injections of increasing intensity, fired similar numbers of APs (Fig. 7*E*; mixed-model ANOVA, effect of treatment *F*_(1,30)_ = 1.65, *p* = 0.21).

**Table 4.** Intrinsic electrophysiological properties of monophasic/putative anaxonic DA cells

	Ctrl (mean ± SEM, [*n*])	Occl (mean ± SEM, [*n*])	Test type, *p* value
**Passive properties**			
Membrane capacitance (pF)	19.17 ± 2.18, [15]	20.81 ± 1.77, [12]	MW, 0.37
Resting membrane potential (mV)	−77.87 ± 1.92, [15]	−70.50 ± 2.49, [12]	*t*, 0.03
Input resistance (mΩ)	960 ± 272, [15]	694 ± 223, [12]	MW, 0.21
**Action potential properties**			
Threshold (pA)	129.7 ± 19.2, [13]	160 ± 29.23, [11]	*t*, 0.38
Threshold (mV)	−30.47 ± 1.09, [13]	−30.70 ± 1.37, [11]	MW, 0.86
Maximum voltage reached (mV)	19.55 ± 2.42, [13]	23.19 ± 1.78, [11]	*t*, 0.26
Peak amplitude (mV)	50.01 ± 2.35, [13]	53.89 ± 2.89, [11]	MW, 0.22
Width at half-height (ms)	0.54 ± 0.03, [13]	0.55 ± 0.03, [11]	*t*, 0.80
Rate of rise (max dV/dt, mV*ms)	240.7 ± 15.82, [13]	254.8 ± 19.63, [11]	*t*, 0.58
Onset rapidness (1/ms)	3.94 ± 0.29, [13]	3.23 ± 0.20, [11]	*t*, 0.06
AHP (mV)	−54.39 ± 1.44, [14]	−54.83 ± 1.34, [12]	*t*, 0.83
AHP relative to threshold (mV)	24.58 ± 1.27, [14]	25.87 ± 1.49, [12]	*t*, 0.51
**Repetitive firing properties**			
Rheobase (pA)	61 ± 19, [14]	86 ± 20, [12]	*t*, 0.23
Maximum no. of APs	10 ± 2, [14]	7 ± 2, [12]	MW, 0.16
First AP delay (ms)	169.2 ± 38.99, [14]	91.34 ± 21.92, [12]	*t*(W), 0.10
Interspike interval CV	0.28 ± 0.04, [14]	0.26 ± 0.04, [11]	*t*, 0.72

Data are mean ± SEM values of passive, AP, and repetitive firing properties for Ctrl and Occl monophasic/putative anaxonic DA cells. Statistical differences between groups were calculated with an unpaired *t* test for normally distributed data (*t*), with Welch's correction (*t*(W)), or with a Mann–Whitney test for non-normally distributed data (MW).

Statistically significant difference.

Overall, in putative anaxonic/monophasic DA cells, the decreases in c-fos and TH expression observed after 24 h naris occlusion are not accompanied by any significant alterations in intrinsic excitability.

### DA cells equipped with an axon shorten their AIS and decrease their intrinsic excitability in response to 24 h naris occlusion

Far less abundant than their anaxonic neighbors, axon-bearing DA neurons tend to have a large soma, and dendrites that branch more widely within the glomerular layer (Galliano et al., 2018). Similarly to anaxonic DA cells, they respond to 24 h naris occlusion by decreasing cFos and TH expression (Figs. 3, 6), but they lack a key characteristic of the former: the dramatic whole-cell structural plasticity, which is the ability to regenerate throughout life. Instead of undergoing lifelong neurogenesis, axon-bearing OB DA cells are exclusively born during early embryonic stages (Galliano et al., 2018). However, we have previously shown that, *in vitro*, this DA subtype can undergo a much subtler type of structural plasticity in the form of AIS alterations. In particular, 24 h reduced activity in the presence of TTX was associated with decreased AIS length in this cell type (Chand et al., 2015). We therefore set out to investigate whether similar AIS plasticity also occurs *in vivo* in response to the same duration of sensory deprivation.

**Figure 8.**
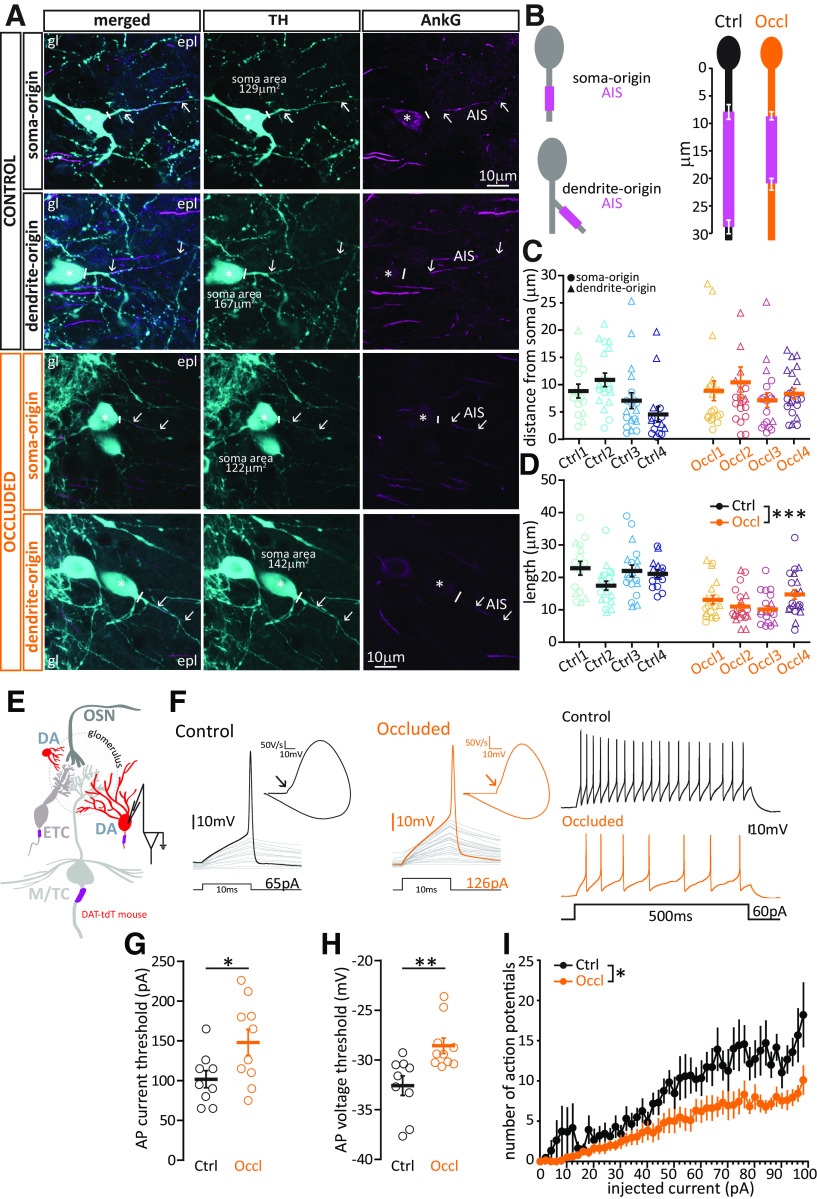
Brief unilateral naris occlusion results in shorter AISs and decreased intrinsic excitability in biphasic/putative axon-bearing DA cells. ***A***, Example average intensity projection images of bulbar axon-bearing DA cells, visualized via staining for TH and the AIS marker AnkG in Ctrl and Occl mice. DA AISs can be found either on a process originating directly from the soma (soma-origin), or on a process separated from the soma by one or more nodes (dendrite-origin). gl, Glomerular layer; epl, external plexiform layer. Solid line indicates the emergence of the axonal process from the soma (asterisk). Arrows indicate AIS start and end positions. ***B***, Left, Schematic representation of soma-origin and dendrite-origin AISs. Right, Mean ± SEM AIS start and end positions of soma-origin + dendrite-origin AISs for each group. ***C***, AIS distance from soma in DA cells from Ctrl and Occl mice. For clarity, one outlier for distance from soma (62 µm, Occl group) is not included in the figure, but is included in all averages and analyses. ***D***, AIS length in Ctrl and Occl mice. ***C***, ***D***, Empty symbols represent individual cells. Different colors represent different mice. Circles represent soma-origin AISs. Triangles represent dendrite-origin AISs. Thick lines indicate mean ± SEM. ***E***, Diagram of whole-cell recordings from large fluorescent cells in DAT-tdTomato mice. ***F–I***, All conventions are the same as in Figure 4*F–I*. *p < 0.05. **p < 0.01. ***p < 0.001.

As for AIS analysis in excitatory neurons, we performed immunohistochemistry in fixed slices of juvenile C57BL/6 mice, double-stained for TH to identify DA neurons and AnkG to measure AISs (AnkG, Fig. 8*A*). A current leitmotiv in the biology of DA neurons is their striking heterogeneity (Zhang et al., 2007; Henny et al., 2012; Chand et al., 2015; Morales and Margolis, 2017; Romanov et al., 2017; Kosaka et al., 2020); and in OB DA cells here, this was also evident in the structure and location of their AIS. We found that OB AISs are of reasonably consistent length (coefficient of variation [CV] = 0.34 in Ctrl cells) but can be situated at highly variable distances from the soma (Ctrl CV = 0.75). Contrary to findings in midbrain DA cells (González-Cabrera et al., 2017; Meza et al., 2018) and in OB dissociated cultures (Chand et al., 2015), we found no consistent relationship between these parameters in bulbar DA neurons (Spearman coefficient of AIS length vs soma distance: Ctrl, *r* = 0.03, *n* = 68 cells, *p* = 0.78; Occl, *r* = 0.04, *n* = 80 cells, *p* = 0.73). We also noted that the AIS of an OB DA neuron can be located either on a process that directly emanates from the soma (“soma-origin” AIS) or on a process separated from the soma by one or more branch nodes (“dendrite-origin” AIS; Fig. 8*A*,*B*) (Thome et al., 2014; González-Cabrera et al., 2017; Höfflin et al., 2017; Houston et al., 2017; Yang et al., 2019; Kosaka et al., 2020). While this peculiar axonal arrangement challenges the traditional view on neuronal input-output transformation (Kaifosh and Losonczy, 2014), it is not unique to bulbar DA neurons. Indeed, midbrain DA neurons have been shown to carry “dendrite-origin” AISs (González-Cabrera et al., 2017; Yang et al., 2019); and recently, the overall variability in AIS length and location in these neurons has been proposed to play a key role in the maintenance of an appropriate pacemaking rhythm in the context of variable dendritic branching (Moubarak et al., 2019). Moreover, “dendrite-origin” AISs are not exclusive to DA neurons: common in invertebrates (Triarhou, 2014), they have also been described in cat and mouse cortex (Meyer and Wahle, 1988; Hamada et al., 2016; Höfflin et al., 2017), in hippocampal pyramidal cells (Thome et al., 2014), and in cerebellar granule cells (Houston et al., 2017).

Occlusion did not affect the proportion of soma- versus dendrite-origin AISs among the OB DA axon-bearing population (soma: Ctrl *n* = 37, Occl *n* = 47; dendrite: Ctrl *n* = 31, Occl *n* = 33; Fisher's exact test for proportions Ctrl vs Occl, *p* = 0.62), nor did it affect the distance of the AIS start position from the soma, independent of axon origin (Fig. 8*C*, different symbols indicate axon origin; Ctrl, mean ± SEM, 7.91 ± 0.73 µm, *n* = 68 cells, *N* = 4 mice; Occl, 8.47 ± 0.94 µm, *n* = 80 cells, *N* = 4 mice; mixed-model ANOVA of log-transformed AIS distance nested on mouse, effect of treatment *F*_(1,13)_ = 1.87, *p* = 0.19; effect of axon origin, *F*_(1,142)_ = 0.65, *p* = 0.42; effect of interaction, *F*_(1,142)_ = 1.94, *p* = 0.17). We did, however, find a sizeable and consistent activity-dependent difference in AIS length, with AISs in Occl DA neurons being significantly shorter than those in Ctrl cells (Fig. 8*D*; Ctrl, mean ± SEM, 20.74 ± 0.84 µm, *n* = 68 cells, *N* = 4 mice; Occl 12.29 ± 0.66 µm, *n* = 80 cells, *N* = 4 mice; mixed-model ANOVA nested on mouse, effect of treatment *F*_(1,24)_ = 93, *p* < 0.0001; effect of axon origin *F*_(1,145)_ = 0.74, *p* = 0.39; effect of interaction, *F*_(1,145)_ = 0.49, *p* = 0.49). In a subset of AnkG-labeled tissue where interslice variability was minimized with histological co-embedding (Fig. 6*G*), AIS shortening in response to brief sensory deprivation was not accompanied by any significant change in the relative intensity of AnkG staining (Ctrl mean ± SEM, 0.75 ± 0.048, *n* = 11; Occl 0.88 ± 0.055, *n* = 12; *t*_(21)_ = 1.74, *p* = 0.10), nor were AIS length and relative AnkG staining intensity significantly correlated (Pearson *r* = 0.21, *n* = 16, *p* = 0.44). We also found no significant correlation between AIS length and relative TH intensity (Pearson *r* = −0.21, *n* = 16, *p* = 0.44), suggesting that the signaling pathways and cellular mechanisms underlying these two pathways in axon-bearing OB DA cells may be reasonably independent (Cigola et al., 1998; Chand et al., 2015).

One key function of the AIS, which houses voltage-activated sodium channels at high density, is to initiate APs (Kole et al., 2007). Previous experimental evidence (*e.g.*, Kuba et al., 2010; Evans et al., 2015) and computational models (see, *e.g.*, Gulledge and Bravo, 2016; Hamada et al., 2016; Goethals and Brette, 2020) have shown that alterations in AIS length, all else being equal, are associated with decreases in neuronal excitability. So does the experience-dependent decrease in AIS length we observe in axon-bearing DA cells correlate with a reduced ability to fire APs? To test this prediction, we again turned to whole-cell patch-clamp recordings in DAT-tdTomato mice, but this time we targeted red cells with a large soma (Fig. 8*E*), and used the biphasic nature of their AP phase plots as a proxy for the presence of an AIS (see Materials and Methods) (Bean, 2007; Chand et al., 2015; Galliano et al., 2018). We found that, with no difference in key passive properties, such as resting membrane potential and membrane resistance (Table 5), putative axon-bearing/biphasic DA cells recorded in acute slices obtained from Occl mice needed more current to reach threshold to generate an AP (Fig. 8*G*; Ctrl mean ± SEM, 102 ± 11 pA, *n* = 9 cells; Occl, 148 ± 16 pA, *n* = 10 cells; unpaired *t* test, *t*_(17)_ = 2.30, *p* = 0.035), and they did so at a more depolarized membrane voltage (Fig. 8*H*; Ctrl mean ± SEM, −32.58 ± 0.99, *n* = 9 cells; Occl −28.57 ± 0.78, *n* = 10 cells; unpaired *t* test, *t*_(17)_ = 3.23, *p* = 0.005). Moreover, when challenged with 500-ms-long current injections of increasing amplitude, Occl DA cells fired fewer APs overall than Ctrl DA cells (Fig. 8*I*; mixed-model ANOVA, effect of treatment, *F*_(1,31)_ = 6.89, *p* = 0.013).

**Table 5.** Intrinsic electrophysiological properties of biphasic/putative axon-bearing DA cells

	Ctrl (mean ± SEM, [*n*])	Occl (mean ± SEM, [*n*])	Test type, *p* value
**Passive properties**			
Membrane capacitance (pF)	22.07 ± 2.21, [11]	21.72 ± 2.07, [10]	*t*, 0.91
Resting membrane potential (mV)	−74.27 ± 2.94, [11]	−77.50 ± 1.73, [10]	MW, 0.65
Input resistance (mΩ)	573 ± 115, [11]	631 ± 117, [10]	MW, 0.46
**Action potential properties**			
Threshold (pA)	102 ± 11, [9]	148 ± 16, [10]	*t*, 0.035
Threshold (mV)	−32.58 ± 0.99, [9]	−28.57 ± 0.78, [10]	*t*, 0.005
Maximum voltage reached (mV)	17.61 ± 3.96, [9]	19.87 ± 3.61, [10]	*t*, 0.68
Peak amplitude (mV)	50.17 ± 4.63, [9]	48.43 ± 3.60, [10]	*t*, 0.76
Width at half-height (ms)	0.50 ± 0.04, [9]	0.53 ± 0.03, [10]	*t*, 0.55
Rate of rise (max dV/dt, mV*ms)	250 ± 31, [9]	227 ± 17, [10]	*t*, 0.51
Onset rapidness (1/ms)	8.22 ± 1.66, [9]	6.63 ± 1.39, [10]	MW, 0.72
AHP (mV)	−55.13 ± 1.50, [11]	−54.27 ± 2.71, [10]	MW, 0.55
AHP relative to threshold (mV)	24.46 ± 1.30, [11]	25.17 ± 2.64, [10]	MW, 0.32
**Repetitive firing properties**			
Rheobase (pA)	32 ± 13, [11]	25 ± 5, [10]	MW, 0.73
Maximum no. of APs	21 ± 4, [11]	15 ± 3, [10]	*t*, 0.23
First AP delay (ms)	273 ± 45, [11]	188 ± 50, [10]	*t*, 0.22
Interspike interval CV	0.24 ± 0.03, [10]	0.22 ± 0.06, [9]	MW, 0.45

Data are mean ± SEM values passive, AP, and repetitive firing properties for Ctrl and Occl biphasic/putative axon-bearing DA cells. Statistical differences between groups were calculated with an unpaired *t* test for normally-distributed data (*t*) or with a Mann–Whitney test for non-normally distributed data (MW).

Statistically significant difference.

In summary, among the OB cell types we analyzed, axon-bearing DA interneurons are the only group that respond to brief, naturally relevant sensory deprivation with a combination of biochemical (Fig. 6*G*), morphological (Fig. 8*D*), and intrinsic functional (Fig. 8*G–I*) plastic changes.

## Discussion

Our results demonstrate that, in young adult mice, brief 24 h sensory deprivation via the unilateral insertion of a custom-made naris plug is minimally invasive yet sufficient to downregulate activity in OB circuits. In response to this naturally relevant manipulation (Fokkens et al., 2012), we find that a very specific subtype of local inhibitory interneurons, axon-bearing DA cells located in the glomerular layer, respond with activity-dependent structural plasticity at their AIS and coincident changes in their intrinsic excitability.

### Can we use structure to predict function *in vivo*? AIS properties and neuronal excitability

Whether on a canonical soma-origin axon or one that emanates from a dendrite, the AIS's structural properties (distance from soma and length) can have a major impact on a neuron's excitability. For the property of AIS position, the precise nature of this impact remains unresolved, and is likely to depend on various factors, including variation in neuronal morphology (Parekh and Ascoli, 2015; Gulledge and Bravo, 2016; Hamada et al., 2016; Goethals and Brette, 2020; Verbist et al., 2020). In contrast, changes in AIS length have a much clearer corollary. Experimental and theoretical results are in close agreement that, all else being equal, a shorter AIS leads to decreased excitability (Kuba et al., 2010; Evans et al., 2015; Gulledge and Bravo, 2016; Sohn et al., 2019; Goethals and Brette, 2020; Pan-Vazquez et al., 2020; Werginz et al., 2020; Jamann et al., 2021). Our data showing brief sensory deprivation-induced AIS shortening and decreased excitability in OB DA neurons are entirely consistent with this coherent picture.

Importantly, while activity-dependent changes in both AIS position and length have been described in cultured neurons (Grubb and Burrone, 2010; Evans et al., 2013, 2015; Muir and Kittler, 2014; Chand et al., 2015; Horschitz et al., 2015; Wefelmeyer et al., 2015; Dumitrescu et al., 2016; Lezmy et al., 2017; Sohn et al., 2019; Booker et al., 2020), plasticity of AIS position without any accompanying length change has yet to be described in intact networks. Indeed, to date, all activity-dependent AIS plasticity described *in vivo* or in *ex vivo* acute slices seems to express itself as length changes (Fig. 8) (Kuba et al., 2010; Höfflin et al., 2017; Del Pino et al., 2020; Pan-Vazquez et al., 2020; Jamann et al., 2021). Failure to describe *in vivo* AIS position changes could be because of a physical impediment to moving this macromolecular structure, which is tightly linked to extracellular matrix proteins (Brückner et al., 2006) when the overall 3D circuit structure is in place. Alternatively, *in vivo* AIS positional changes might be possible, but we have yet to probe the cell types that are capable of this with an appropriate manipulation. Finally, it is important to note that the main caveat of most *in vitro* and all *in vivo* AIS plasticity studies is that analysis has been done at the population level, and links between AIS and excitability changes on a cell-by-cell level are few and far between. Future studies will need to address this by pairing electrophysiological recordings with tools for AIS live imaging (Dumitrescu et al., 2016).

### Implications for olfactory processing

We find here that 24 h sensory deprivation leaves bulbar excitatory neurons' intrinsic excitability unchanged, but recruits structural and intrinsic plastic mechanisms in a specialized population of inhibitory interneurons, as well as producing downregulated TH levels in all DA neurons. What are the functional implications of these different neuronal responses? By releasing GABA and dopamine that can target release probability at OSN terminals, DA neurons act as gain controllers at the first synapse in olfaction (Hsia et al., 1999; Borisovska et al., 2013; Vaaga et al., 2017). Thanks to their rapid activity-dependent regulation of TH expression, both subtypes of DA cell might respond to decreased afferent input by producing and releasing less dopamine, thus decreasing feedback inhibition of OSN terminals. This could be a very effective mechanism to rapidly counterbalance the effects of sensory deprivation by increasing the gain of the first synapse in the olfactory system, potentially thereby heightening odor sensitivity. Indeed, our data represent the fastest known description of this extremely well-described phenomenon which, at least following longer-term manipulations, appears responsible for balancing bulbar input-output functions in the face of sensory deprivation (Baker et al., 1993; Wilson and Sullivan, 1995; Cho et al., 1996).

The AIS shortening and decreased excitability in axon-bearing DA cells could further accentuate the deprivation-associated relief of inhibition in the glomerular layer. Decreases in TH levels and decreases in neuronal excitability appear broadly synergistic, and together should locally increase the gain of nose-to-brain transmission. However, dopamine has recently been shown to have complex postsynaptic effects on glomerular circuitry (Liu, 2020), by which any changes in OSN presynaptic inhibition driven by plasticity in local DA cells might be at least partially counteracted. Also, axon-bearing DA cells have widely arborized dendritic trees and a long-spanning axon (Banerjee et al., 2015; Kiyokage et al., 2017; Galliano et al., 2018), and are believed to contribute not only to local intraglomerular signaling and gain control, but also, by means of long-range lateral inhibition (Liu et al., 2013; Whitesell et al., 2013; Banerjee et al., 2015), to odor identification and discrimination (Uchida et al., 2000; Urban, 2002; Linster and Cleland, 2009; Zavitz et al., 2020). Decreasing their excitability might therefore be expected to produce olfactory discrimination deficits. How can we reconcile these two potentially opposing effects? One could speculate that when the network is deprived of sensory inputs, a first, fast-acting response dampening all (intraglomerular and interglomerular) inhibition to increase overall sensitivity (Kuhlman et al., 2013) could be prioritized over maintaining fine discrimination. Then, if the sensory deprivation persists, a more nuanced solution might be implemented in which other neuron types adapt their excitability to reach a new stable network set point (Gainey and Feldman, 2017), while permitting interglomerular connections to reprise their more powerful long-range inhibitory function. In addition, the long-range interglomerular projections of glomerular layer DA neurons have also been proposed to underlie gain control modulation of OSN→M/TC signaling (Bundschuh et al., 2012; Banerjee et al., 2015), so targeted decreases in their excitability could be another mechanism for ensuring maximal impact of diminished OSN inputs, especially in the initial stages once the state of deprivation begins to resolve. In this way, specific plastic changes in one cell type might shift the balance of information processing in sensory circuits to prioritize detection over discrimination when input activity is diminished.

### Homeostasis in cells or circuits? Inhibitory neurons as first responders

While not preponderant in cortex, inhibitory neurons constitute the main population in the OB (Shepherd, 2004). Heterogeneous in all brain areas, inhibitory neurons can be just as plastic as their excitatory counterparts, but can respond differently to the same sensory input (Gainey and Feldman, 2017). Understanding this differential excitatory/inhibitory plasticity and its time course could help unpack one of the most puzzling phenomena in neuroscience: how stability and plasticity coexist to ensure both homeostasis and learning (Fox and Stryker, 2017). Indeed, one could speculate that while the plasticity of excitatory neurons is mostly Hebbian and aimed at supporting the acquisition of new associations (Bekisz et al., 2010; Yiu et al., 2014; Gao et al., 2017), one of the main functions of activity-dependent plasticity in inhibitory neurons is to act as “first responders.” In this scheme, plasticity in local inhibitory cells acts to compensate a short-lived change in sensory input and to maintain homeostasis, not at the single-cell level, but at the network level. If then the sensory perturbation persists and becomes the “new normal,” excitatory cells might need to activate homeostatic plasticity mechanisms and inhibitory neurons to downscale their own fast-acting plastic response, to reach a new network set point while maintaining an appropriate dynamic range (Turrigiano, 2012; Wefelmeyer et al., 2016; Gainey and Feldman, 2017; Keck et al., 2017). The overall circuit response to a changed sensory stimulus cannot thus be inferred by solely looking at principal neurons (Hennequin et al., 2017), or by simple arithmetic sums of plastic changes in the various neuron types, or without appreciation of the length and scope of sensory manipulation. Future studies will need to holistically address how activity-dependent plasticity is differentially expressed in inhibitory and excitatory neurons to shape information processing in distinct brain circuits.
